# Current Progress in Research into Environmentally Friendly Rigid Polyurethane Foams

**DOI:** 10.3390/ma17163971

**Published:** 2024-08-09

**Authors:** Sylwia Makowska, Dawid Szymborski, Natalia Sienkiewicz, Agnė Kairytė

**Affiliations:** 1 Institute of Polymer and Dye Technology, Faculty of Chemistry, Lodz University of Technology, 90-924 Lodz, Poland; sylwia-magdalena.makowska@vilniustech.lt (S.M.); 231438@edu.p.lodz.pl (D.S.); natalia.sienkiewicz@p.lodz.pl (N.S.); 2 Civil Engineering Research Centre, Vilnius Gediminas Technical University, Saulėtekio av. 11, 10223 Vilnius, Lithuania

**Keywords:** polyurethane foam, sustainability, waste fillers, polyols, isocyanates, properties

## Abstract

Polyurethane foams are materials characterized by low density and thermal conductivity and can therefore be used as thermal insulation materials. They are synthesized from toxic and environmentally unfriendly petrochemicals called isocyanates and polyols, which react with each other to form a urethane group via the displacement of the movable hydrogen atom of the −OH group of the alcohol to the nitrogen atom of the isocyanate group. The following work describes the synthesis of polyurethane foams, focusing on using environmentally friendly materials, such as polyols derived from plant sources or modifiers, to strengthen the foam interface derived from plant precipitation containing cellulose derived from paper waste. The polyurethane foam industry is looking for new sources of materials to replace the currently used petrochemical products. The solutions described are proving to be an innovative and promising area capable of changing the face of current PU foam synthesis.

## 1. Introduction

The European Union’s environmental policy boils down to achieving key energy targets set by the European Parliament over the years. One of these was to reduce greenhouse gas emissions by 20 percent by 2020. However, these have turned out to have fallen by 31 percent from 1990 levels and reached a historic low in 30 years. In contrast, in 2019, the European Commission presented the concepts of the European Green Deal, which calls for a 55% reduction in greenhouse gas emissions by 2030 [1,2,3] and to decarbonize the EU economy by 2050, in line with the commitments under the Paris Agreement, which set the EU as the first economy and society in the world to become climate-neutral [4]. Achieving a high decrease in gas emissions in 2020 was a satisfactory result linked to the introduction of fundamental changes, such as exhaust gas cleaning systems, forest management, and appropriate waste management, among others. However, further targets present society with further challenges. One of these is improving the insulation of buildings. The tertiary and residential sectors consume a very large amount of energy to ensure comfortable temperatures in buildings, as such temperatures may be lost due to the use of insufficient thermal insulation. The work presented here discusses issues related to modifications of polyurethane foams that meet European environmental objectives. This type of material should have a sufficiently low thermal conductivity coefficient, meet all building standards, and be developed using environmentally friendly, renewable raw materials [5,6,7,8,9]. Currently, polyurethane foams are derived from toxic chemicals. Their recycling also involves some complications and is not realized to any great extent. It is therefore important to develop polyurethane foam (PUF) using non-toxic and environmentally friendly compounds, while maintaining adequate thermal, acoustic, and mechanical properties.

### Definition of Polyurethanes

In attempting to define polyurethane foams, it is important to determine what characteristics the foam has. It is a two-phase material consisting of a solid and a gaseous part, produced during the foaming process. The gaseous phase is responsible for ensuring a low heat-transfer coefficient, while the solid phase is responsible for ensuring the material’s mechanical strength. Polymer foams can be divided into four types based on pore size:

Macrocells >100 μm,
Microcells $\left(1-100\;\mu m\right),$
Ultracells $\left(0.1-1\;\mu m\right),$
Nanocells $\left(0.1-100\;nm\right)$ [10].

**Figure 1:**
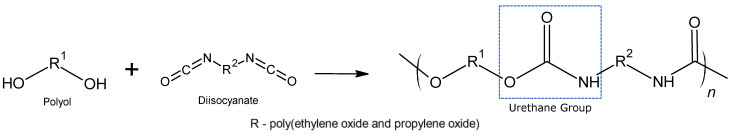
The basic reaction between a polyol and an isocyanate is to form urethane groups.

Polyurethanes (PUs) are linear or cross-linked polymers whose characteristic feature that distinguishes them from other polymers is the presence in their main chain of a urethane group with a [-O-CO-NH-] structure. Polyurethanes are traditionally obtained by the polyaddition of compounds containing two or more isocyanate groups in the molecule (di- or triisocyanates), with compounds containing two or more amine or hydroxyl groups (polyols) [11]. The reaction proceeds by displacement of the movable hydrogen atom of the −OH group of the alcohol to the nitrogen atom of the isocyanate group, with the formation of the bond characteristic of urethanes shown in Figure 1. The reaction is carried out in the company of appropriately selected auxiliary agents.

Polyurethanes (PUs) are a group of compounds in which, depending on the starting composition, raw materials, and methods of obtaining the polymer, the properties of the final material can be influenced. Polyurethanes are segmented polymers, rigid segments comprise aromatic or aliphatic diisocyanates (−NCO) and flexible segments consisting of aliphatic polyols (−OH). Rigid segment isocyanates are formed by reacting toluenediamine (TDA) or methylenediamine (MDA) with a phosphagen to produce toluene diisocyanate (TDI) or methylenediphenyl diisocyanate (MDI) [12]. By modifying the rigid and flexible segments in the structure of the macromolecule, it is possible to influence properties such as the following: mechanical strength, thermal strength, modulus of elasticity (rigid segments), softness, elasticity, elongation at break, and resistance at low temperature (average temperature in the seas and oceans 4 °C; soft segments).

## 2. Materials

The basic raw materials in the synthesis of polyurethanes are suitably selected mixtures of polyols and isocyanates. Usually, several excipients are added to the mixture composition, the most important of which are as follows:

Blowing agents,

Surfactants,

Catalysts,

Antipyrines, fillers, pigments, and dyes [10,12,13,14].

### 2.1. Description of Currently Used Compounds

The versatility of polyurethane foams is based on the possibility of modifying the substrates used to obtain them. Controlling their composition or changing certain chemical properties results in foams with a variety of properties. Their stiffness and flexibility mainly depend on the type and quantity of compounds used during synthesis. PUs consist of polyols and isocyanates forming different structural domains, which are divided into hard- and soft-chain fragments. Their mobility determines material properties such as hardness or flexibility. PU foams are therefore classified, in terms of mechanical properties, into flexible and rigid foams, as well as the less commonly produced semi-rigid and semi-flexible foams. The distinction between them is mainly made by determining the parameter of network density, which is much higher in the case of rigid foams. The highly cross-linked structure contains a higher number of urethane and urea groups, thus inducing strong inter-chain interactions. In addition, rigid PUFs contain a large number of aromatic rings derived from isocyanates, which promote π-π interactions between network segments and increase the rigidity of the structure [13,15].

#### 2.1.1. Polyols

The principal components of polyurethane (PU) compositions are polyols, which make up about two-thirds of the composition of polyurethanes. There are two types of polyols: polyether and polyester. Polyether polyols are synthesized by reacting epoxides with compounds that have an active hydrogen atom. Polyester polyols, on the other hand, are obtained by polycondensation of multifunctional carboxylic acids and polyhydroxy compounds. We classify these compounds according to their properties. Polyethers are generally harder and have very good dynamic properties—they have a lower molecular weight. Polyester polyols, on the other hand, are softer and have a high tensile strength—they have a higher molecular weight. Polyols used as flexible materials are made from raw materials containing a lower number of hydroxyl groups, which are functional groups. Examples of such compounds are dipropylene glycol, which has two hydroxyl groups, or glycerol, which has three hydroxyl groups. In contrast, polyols, which produce foams with higher stiffness, contain a higher number of hydroxyl groups. These include sucrose, with a functionality of eight; sorbitol, with a functionality of six; and toluenediamine, with a functionality of four. There are also other types of polyether polyols. Poly(tetramethylene ether) glycol is obtained by polymerizing tetrahydrofuran for use in high-performance elastomethodic applications [13,16,17,18]. Several properties of foams can be controlled by changing the functionality of the polyol. For example, increasing the functionality of the polyol without changing the molecular weight results in a slight increase in the hardness of the foam and a slight decrease in tensile and tensile strength. In contrast, increasing the molecular weight while maintaining functionality increases the tensile strength and elongation [19]. Higher functionality means that a stronger and more intensely cross-linked structure is created during the cross-linking process due to the higher number of active centers in the material chain [20]. In general, polyols with longer alkyl chains and low functionality produce flexible polyurethane foams, whereas polyols with high functionality and shorter chains produce rigid polyurethane foams due to the formation of a more cross-linked structure [21]. Polyols are used in the form of mixtures of molecules that have a different number of hydroxyl groups. For this reason, the determination of the average functionality of the polyol is required. In industrial applications, polyols have a strictly controlled composition in order to achieve similar properties for the entire production [22]. In summary, the properties of polyurethane are closely related to the polyol from which it is derived. It is therefore important that the polyol offers the performance required for the application in question [23].

#### 2.1.2. Isocyanates

Isocyanates, along with polyols, are the other main components of PUR compositions. They are derivatives of isocyanic acid, with the following general formula:

*R* (*N* = *C* = *O*)*_n_*;

*n* = 2 − 5 (1)

The isocyanate group is highly reactive due to its occurrence in various resonance structures, reacting with numerous chemical groups:

- Reaction with −OH groups—catalyzed by tertiary amines and more strongly by organotin compounds, catalyst selection is of strong importance for foaming, organotin catalysts accelerate reactions with OH more strongly than amines, amines with H_2_O. Organotin catalysts, the most commonly used, are cinnamate and dibutyltin caprylates and laureates.
- The reaction with −NH_2_ groups proceeds faster and does not require catalysts. However, it requires the use of diols having NH_2_ groups or a mixture to control the synthesis reaction.
- By reacting diisocyanates and diepoxy compounds in the presence of suitable porophores and catalysts, it is possible to obtain foamed polyoxazolidones in the presence of suitable catalysts.

**Figure 2:**
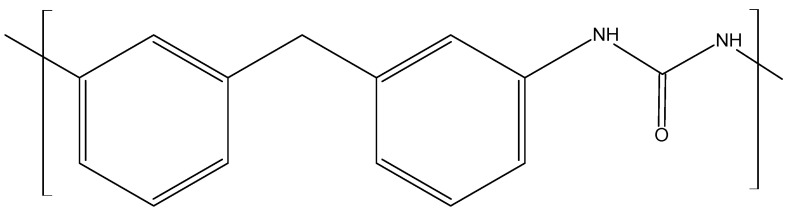
MDI structural formula.

**Figure 3:**
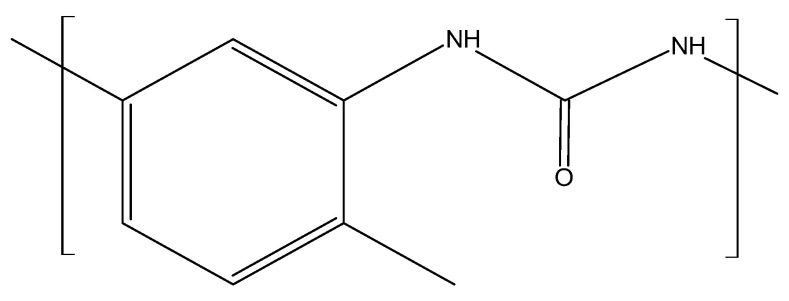
TDI structural formula.

Isocyanates are representatives of a class of compounds that are characterized by their high reactivity and versatility. For this reason, they find a wide range of applications. Their greatest advantage from a chemical point of view is their reactivity with molecules possessing active hydrogen atoms. Such hydrogen is usually found in molecules with alcohol and amine functionalities, as well as in water [24]. Isocyanates can be divided into difunctional or heterofunctional, and aromatic or aliphatic [22]. The most commonly used in PU materials technology are aliphatic isocyanates and aromatic isocyanates, which are used much more frequently than linear isocyanates due to their lower price and greater reactivity. The most commonly used aromatic isocyanates are MDI (Figure 2) and TDI (Figure 3), which account for about 90% of total isocyanate consumption. Aliphatic isocyanates are also used, such as isoprene diisocyanate (IPDI) or hexamethylene diisocyanate (HMDI); these compounds are mainly used in coatings where transparency and color are the key parameters. Polyurethanes based on aromatic isocyanates darken when exposed to light [25]. Polyurethanes made from aromatic isocyanates typically have higher glass transition temperatures and tensile strengths but lower elongation at break and impact strengths, and rigid and thermoset PUs are obtained with them. In contrast, polyurethanes derived from aliphatic isocyanates will have similar properties to rubber materials with higher elongation at break and lower tensile strengths [19,26]. PU coatings are obtained from them. In addition, they are more readily miscible with pigments, thus providing the final product with a glossy appearance and better UV resistance [21].

MDI is used for rigid foams used to make insulation and automotive parts. TDI, on the other hand, is used in the production of flexible polyurethane foams, which are used to make coatings, adhesives, sealants, and elastomers. To obtain MDI, a reaction must be carried out between aniline and formaldehyde, accompanied by hydrochloric acid as a catalyst. This reaction leads to a mixture of methylenedianiline (MDA) and multimeric precursors of MDA. MDA is then treated with phosgene, leading to a mixture of MDI isomers. In contrast, to obtain TDI, the reaction of toluene with nitric acid must be carried out to obtain diaminotoluene (TDA). TDA is then reacted with phosgene to obtain TDI [12,13,17]. Most isocyanates have two isocyanate groups, with the exception of compounds such as diphenylmethane diisocyanate, which consists of mixtures of different molecules that contain two or more isocyanate groups. Therefore, this compound has an average functionality of 2.7. It is possible to carry out modifications to isocyanates to change parameters such as volatility or the freezing point. As a result, chemical processes involving them become easier to carry out, and the final polymers produced have improved properties [22]. Both TDI and MDI were subjected to scrutiny regarding health issues. Toxicological studies on humans and animals have shown that the compounds tested can cause asthma in sensitive individuals even at very low concentrations. As a result, they are listed in various lists regulating hazardous chemicals [27]. This leads to a search for substitute compounds with similar properties and low or no toxicity.

#### 2.1.3. Blowing Agents

In the production of polyurethane foams, it is necessary to create the porous structure of the material using blowing agents. Two types of compounds are used:

- Physical blowing agents, such as solvents with a low boiling point: pentane, acetone, or hexane. They form pore structures during evaporation.
- Chemical blowing agents, such as water, which expand the polymer by producing carbon dioxide [19].

CO_2_ is currently the most commonly used foaming agent to create polymer foams, especially polyurethane foams [10].

In discussing blowing agents, it is important to mention the types of porous structures formed during foaming processes. Gases have a much lower thermal conductivity than solids or liquids due to the larger distances between molecules. The introduction of a pore-enclosed gas is therefore associated with a reduction in heat conduction through the solid. Pores trapped in a solid matrix can be divided into two types: open pores, which are connected, or closed pores, which are separated by a solid phase. To achieve a thermal conductivity lower than that of still air, a porous structure comparable to the so-called average free path of air (~70 nm, 300 K, and 1 atm) must be achieved. One example of this phenomenon is an aerogel made from nanocellulose, which has a pore size of approximately 30 nm and a porosity of 0.989. It exhibits a thermal conductivity of 0.018 W/mK, lower than that of stationary air. However, the formation of such an extensive porous structure is associated with a reduction in the service strength of the material, which can pose a significant problem in its applicability, e.g., in construction [28,29].

#### 2.1.4. Surfactants

The final properties of the foam depend on the quality of the rigid foam structure and the number and size of gas bubbles that have formed during the foaming process. The most common surfactant is a copolymer made of silicone and strains of poly(ethylene oxide and propylene oxide) [30]. Their use significantly alters product properties. Scientific studies have shown that surfactants do not change the reaction kinetics in the polyurethane foaming process, but they do affect the bubble generation process and their stabilization in the foam structure [31]. The cell size in the foam produced is highly dependent on the structure of the silicone surfactant. Compounds with a higher silicone content provide a lower surface tension, thus helping to increase the number of bubbles introduced during the mixing process. Consequently, a product derived from a surfactant with a higher silicone content will have a smaller bubble size. The main role of silicone surfactants in the synthesis of rigid polyurethane foams is to control the size of gas bubbles, ensuring an optimal fine-cell structure with a narrow cell size distribution [19,30].

#### 2.1.5. Catalysts

As the definition of a catalyst shows, it causes the initiation of a reaction. In the case of foams, it affects the reaction balance between isocyanates and polyols and isocyanates and foaming agents. Imbalances can, among other things, cause the foam to collapse or form inappropriate cells, which can be closed or opened prematurely. Amine and metalorganic compounds are common in the industry. Amine catalysts are better at catalyzing the isocyanate-foaming agent reaction, while organometallic catalysts will affect the isocyanate–polyol reaction [19,32].

#### 2.1.6. Flame Retardants

Despite the numerous advantages and applications of polyurethane foams, their high flammability and ability to spread fire quickly is a major concern. Several attempts have been made to make foams fire-resistant. The high flammability is caused by the porous structure, which facilitates the diffusion of oxygen in the foam matrix. In addition, pure PU has a low LOI (limited oxygen index) of 18%. Halogenated compounds are highly effective in reducing the flammability of PU, but they pose a high health and environmental risk, thus leading to further research. Inorganic compounds (e.g., aluminum hydroxide and magnesium hydroxide), nanomaterials (graphene and expandable graphite), and siloxanes significantly improve the flame retardancy of PU foams. [33,34].

### 2.2. Fillers for PU Foams

**Figure 4:**
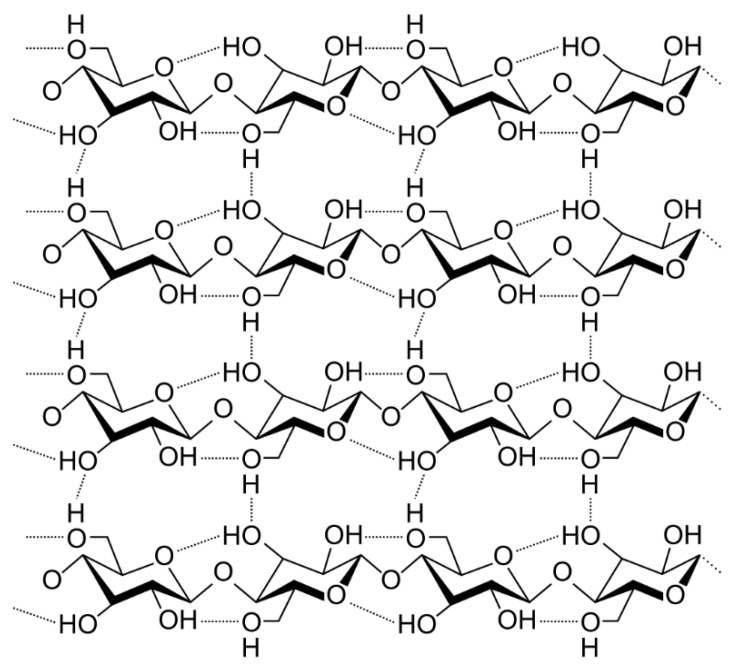
Cellulose-chain fragment.

To meet the environmental objectives of the European Union, polyurethane foams can be filled with waste materials that not only behave inside the foam matrix as inactive fillers but can also act to reinforce the material. Wheat hulls, eucalyptus fibers, or various types of wood consist of cellulose (Figure 4), which is an unbranched biopolymer, a polysaccharide made up of 3000–14,000 D-glucose molecules linearly linked by β–1.5-glycosidic bonds [35]. These contribute to the formation of rigid long strands, which are arranged in parallel and form micelles connected by hydrogen bridges. The described chains have a length of approximately seven micrometers.

Cellulose, in its chemical structure, contains hydroxyl groups on the surface, which are theoretically able to react with isocyanates in a similar way to the reactions occurring between polyols. This process should have a reinforcing effect on the foam composite interface. This property offers the possibility of improving the mechanical properties of the foams, in turn opening the way for lowering the isocyanate concentration, while maintaining good physical properties. This presents significant opportunities for the use of cellulosic biomass in polyurethane foam matrices. Numerous scientific publications have been produced describing the various modifiers. The materials used to modify foams include wheat hulls, rice hulls, or waste from coffee production. These materials most often end up in landfills, so it is financially beneficial to use them in foam production. Previous research has demonstrated the use of cellulose nanofilaments with improved tear resistance and stiffness. The results showed that they are a very good modifier for foams, while they cannot be widely used due to their high cost and difficult processing compared to ground coffee waste or husks [36,37,38,39]. The cited scientific publication also shows the negative effect of using modifiers of this type. The authors, using rapeseed and rice straw for rigid polyurethane foams with a high isocyanate index, noted that higher modifier concentrations could be detrimental to structural stability during foaming, leading to partial collapse of the material [40]. Therefore, an important aspect is the development of a composition containing the right amount of modifier and also with a reduced isocyanate content. The proper preparation of the biomaterial is also an important issue. Particle size and specific surface area have a significant impact on the final properties of the product, which can be obtained via the proper milling of the material and a subsequent sieve analysis to obtain precisely defined particles. Another aspect is also the availability and activity of hydroxyl groups in the cellulose structure. Cellulose waste may be contaminated with various types of groupings, the removal of which may be necessary to obtain a satisfactory performance of the foam product. The drying process is also an important parameter, due to the often-high moisture content of such materials that makes them unfavorable in the synthesis of polyurethane foams.

A good source of bio-based materials containing cellulose particles is the paper industry. The economy produces very large amounts of wastepaper, used packaging, or cardboard, which, after preparation, can be used in the production process. Of course, the possible influence of dyes or other substances present in paper waste must be kept in mind and taken into account, which means that the material must be modified. One way is to use trichloroacetic acid and isopropyl alcohol. [41,42]. Paper is very commonly subjected to bleaching processes with compounds containing chlorine, which can attach to the cellulose structure and be removed by using, among other things, sodium hydroxide in the appropriate concentration.

NaOH modification is used in the paper industry to remove hemicellulose from the fiber surface and can lead to the exposure of hydroxyl groups. This process is called mercerization.

## 3. PU Foam Production

The foam structure is formed by the growth of gas bubbles in the polymer matrix. Initially, the density of the foam decreases slightly, and small dispersed spherical gas bubbles form in the liquid matrix. In the next stage, an increase in closed bubbles follows. Subsequently, the formation of open-cell foam is observed by breaking down the cell walls. The ratio of open to closed cells is crucial to the properties of the foams. If the cells are not open at the end of the expansion phase, the foam collapses, because the rapid diffusion of carbon dioxide (CO_2_) out of the cells is much faster than that of the air [10,43]. Pore size is also of particular importance in the final properties of the material.

The process of obtaining PU foams from classically used materials involves several stages, of which the following can be distinguished:

- The latent period, which lasts from the moment the components are mixed until the mixture starts to grow in volume.
- The growth period, which lasts from the moment the volume of the mixture begins to increase until it reaches its highest volume; here, an exothermic polymerization reaction takes place, causing the low-boiling liquids to evaporate, and the gas fluffs up the mixture while it is still in a plastic state, giving it a cellular structure.
- Stabilization (gelation) period, in which the foamed mixture is transformed into a property-stable plastic; the inherent reaction and side reactions of allophane and biuret bond formation still occur during this period.
- The maturation period of the foam, during which all ongoing chemical reactions take place to completion; the structure is finally established; and the properties, shape, and size of the foam are determined (this period generally lasts up to several hours).

Theoretically, when conducting PU synthesis, we should use equal amounts of diisocyanate/polyisocyanate and diol/polyol to achieve the full degree of polymerization. In practice, a small amount of polyisocyanate is used due to compensate for its loss to the reaction of the −NCO group with the −OH group of water, which is available in the polyol. In addition, the −NCO group reacts with other functional groups, for example, amines, urea, or carboxylic acids. The modification of the composition and the wide range of possible chemical reactions leads to polyurethane with different properties and degrees of cross-linking. A highly cross-linked polymer network leads to thermoset PUs that are thermostable and suitable for applications at relatively high temperatures (up to 60 °C). In contrast, a high degree of cross-linking leads to limitations in the recycling of polyurethanes, as mentioned later in this paper [33,44].

The PU foam production technology involves mixing all the components, except the isocyanate, which is added at a later stage. An unwanted chemical in the synthesis is water due to side reactions; however, a significant amount of water is unavoidable in the synthesis of flexible PU foams. Water reacting with isocyanate generates urea and carbon dioxide, which is the most common blowing agent in polyurethane foams, and the amount of water depends on the amount of water present in the reaction mixture [33,44].

## 4. Properties of Polyurethane Foams

### 4.1. Chemical Properties

Polyurethane foams are usually characterized as chemically inert and generally non-toxic compounds. However, they are treated as combustible materials and can pose a risk during a fire. In addition, the foam combustion process produces large amounts of carbon monoxide, nitrogen oxides, hydrogen cyanide, and other toxic gases. This makes it necessary to modify foams to reduce their flammability [14,20,34]. Some of the most common solutions are the introduction of suitable modifications or the use of additives to reduce flammability. Compounds such as phosphorus, halides, or nitrogen are mainly used. Satisfactory results are also obtained with the use of clays [45].

### 4.2. Physical Properties

Polyurethane foams have good mechanical properties depending on whether we are analyzing rigid or flexible foams. More flexible foams are lightweight, durable, and easy to shape. Rigid foams, on the other hand, are characterized by high tensile strength, tear resistance, and abrasion resistance, which gradually increases with the hardness of the foam. They are characterized by low thermal conductivity, which depends mainly on the thermal conductivity of the gas trapped in the foam cells. In addition, polyurethane foams have good resistance to mineral oils, grease, petrol, and organic solvents. They are also resistant to acidic and alkaline solutions. They have sorptive properties. When PUR is made from aromatic isocyanates and exposed to UV light, yellowing of the material is observed due to the content of chromophores that interact with light. This process does not lead to the deterioration of the material’s physical properties [20,46].

Good vibration-damping properties are another characteristic of PU foams. Sound waves striking the structure of the material and passing through the material induce stresses in the cell walls that force them to twist. As a result, the entrance or exit of sound is easily controlled [46].

## 5. Recent Advances in the Production of PUR Foams

Recently, there has been an increase in the impact of the concept of sustainable development, which includes environmental activities connected with reducing the use of petrochemical-derived fuels and converting post-production feedstock into raw materials for the synthesis of polymeric materials [47,48]. In 2019, the global PUR market based mainly on petrochemical feedstock was valued at USD 95.13 billion and is expected to reach USD 149.91 billion by 2023. [49]. As a result, intensive efforts are being made to reduce the use of fossil fuels and replace them with environmentally friendly solutions. In this regard, the development of bio-based PUR foams can be performed in two ways—(i) by limiting the use of petrochemical fuels and replacing them with polyols of natural origin sourced from natural raw materials and (ii) via the modification of PUR foams with the addition of plant or waste fillers [50].

### 5.1. Rigid PUR Foams Derived from Bio-Based Polyols

**Figure 5:**
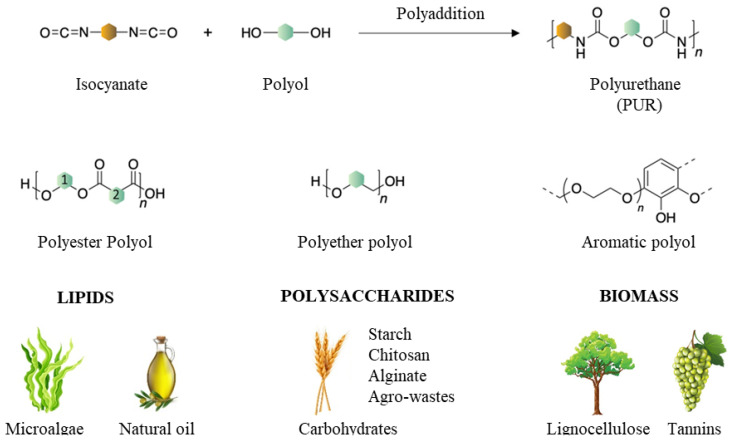
Bio-based polyols derived from diverse biomass resources.

One way to meet sustainable development is to introduce bio-polyols based on raw materials of natural origin into the synthesis of PUR [51]. Due to the wide variety of renewable raw materials, it is possible to obtain bio-polyols with different chemical structures—for example, lipids are used for the synthesis of polyester polyols, polysaccharides are used for obtaining polyether polyols, and lignocelluloses are used for the synthesis of aromatic polyols (Figure 5) [52]. Previous studies have shown that the use of bio-polyols derived from natural raw materials results in the production of PUR foams with physical and mechanical properties comparable to PUR foams synthesized from petrochemical-based polyols [51,52,53,54,55].

**Figure 6:**
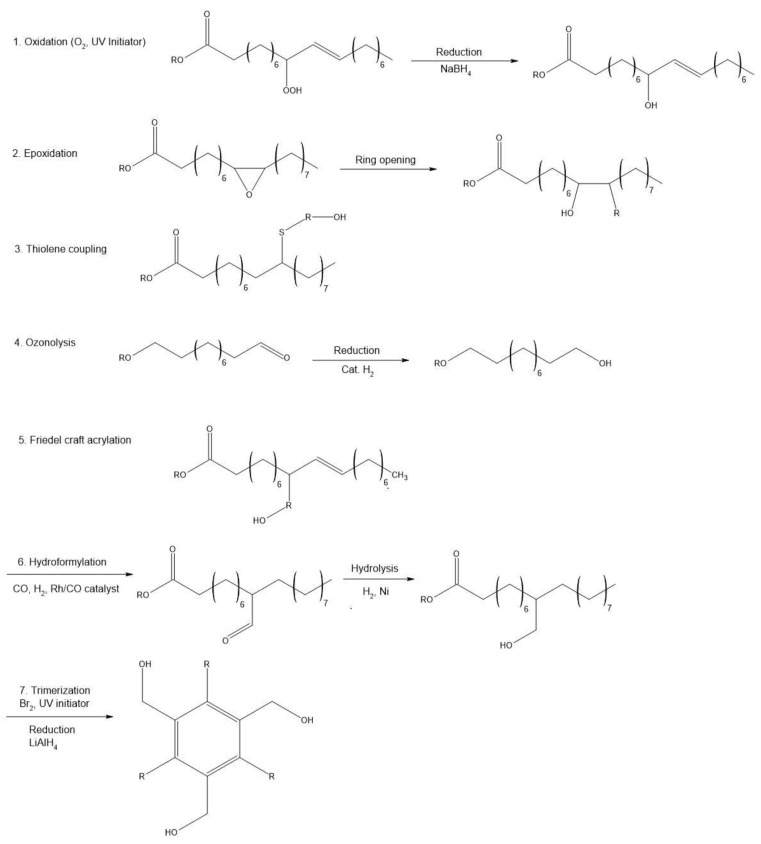
Modifications of vegetable oils for the polyurethane foam industry.

A feature that makes it possible to use oils in the production of polyols is the presence in their structure of hydroxyl groups with different availability and activity. In order to increase their functionality, it is necessary to carry out modifications through, inter alia, epoxidation, hydroformulation, ozonolysis, transesterification, or amidation [23]. Figure 6 shows the most preferred methods of chemical modification of oils. Studies show that epoxidation is the most preferred process for hydroxyl functionalization [56,57]. It is a method that generates low production costs and requires low-cost chemical reagents. The conversion of the double bond into an epoxy ring is carried out using various methods, such as in situ epoxidation using peracids (peracetic acid and perbenzoic acid) in the presence of an acid catalyst. Another method is epoxidation of the double bond with organic and inorganic peroxides, including transition metal catalysts, or epoxidation with halohydrins using hypohalic acids and their salts and epoxidation with molecular oxygen [58]. Hydroxyl groups play an important role in the thermal conductivity of foams, as they react more rapidly in the foaming reaction compared to secondary groups. This creates a weak three-dimensional network, which is poor at retaining the gas produced during the foaming reaction [59].

Several researchers have synthesized bio-polyols by using different vegetable oil and lignocellulosic materials to develop environmentally friendly rigid PUR foams.

#### 5.1.1. Soybean Oil-Based Polyols

Petrovic et al. synthesized two groups of bio-polyols based on soybean oil. The development of bio-polyol synthesis involved two methods—ozonolysis and hydroformylation of unsaturated groups of soybean oil, respectively [60,61]. Subsequently, such developed bio-polyols were used for the production of rigid PUR foams, which showed thermal insulating and mechanical properties comparable to their fossil-based analogs. Similar results were reported in the work of Tan et al. [62]. Rigid PUR foams synthesized based on soybean oil-based polyol maintained a well-developed cellular structure, with a high content of closed-cells, as well as comparable thermal insulating properties. The impact of the incorporation of epoxidized soybean oil-based polyol on the properties of PUR foams was also investigated in the work of Tu et al. [63]. Bio-polyols with different isocyanate index values (in the range of 50–100) were used for the synthesis of PUR foams. It was found that when the isocyanate index was lowered to 60, the density of the foams was slightly reduced; however, when the isocyanate index was decreased to 50, the density significantly increased. Ji et al. [64] investigated the impact of the replacement of petrochemical polyols with soy-based polyols on the physicomechanical properties of PUR foams. The bio-polyols were synthesized in a two-step reaction that involves the epoxidation of soybean oil and oxirane ring opening with different compounds—(i) methanol, (i) phenol, and (iii) cyclohexanol. PUR foams with bio-polyol with methanol exhibited a more uniform structure, the smallest cell size, the greatest density, and the highest thermal conductivity. The incorporation of 25 wt.% of phenol resulted in the synthesis of PUR foams with improved mechanical properties, glass transition temperature, and thermal stability. At 50 wt.% of polyol content, the physicomechanical properties of PUR foams deteriorated. The authors reported that this may be connected with the low cross-linking density of foams, as a result of the presence of excessive benzene rings and high steric hindrance. The addition of bio-polyol with cyclohexanol resulted in the deterioration of the mechanical performances of PUR foams due to the plasticizing effect of six-membered rings. In another work, Narine et al. [65] investigated the impact of different bio-polyols on the physicomechanical properties of PUR foams. The bio-polyols were synthesized via the ozonolysis of canola oil and hydrogenation of soybean and crude castor oil. The authors investigated the impact of three different bio-polyols with different positions of hydroxyl groups in a molecule and the impact of a dangling chain of bio-based polyols on the properties of the foams. It was found that PUR foams synthesized from canola-based polyols showed better mechanical properties compared to PUR foams containing soy-based and castor-based polyols. This was attributed to several factors, such as differences in the position of hydroxyl groups, different numbers of hydroxyl groups, and different lengths of dangling chains that affected the reactivity and foaming kinetics of the reaction mixtures.

#### 5.1.2. Rapeseed Oil-Based Polyols

Similar results were reported in the work of Kurańska et al. [66]. The authors investigated the impact of the presence of secondary hydroxyl groups in rapeseed oil-based polyol molecules on the reactivity of a reaction mixture. The results of dielectric polarization and maximum temperature measured during the foaming process confirmed that the replacement of petrochemical polyol by bio-based polyol decreased the reactivity of the PUR systems. The incorporation of rapeseed oil-based polyol into PUR formulations was also investigated by Zieleniewska et al. [67]. The replacement of petroleum-based polyol resulted in the formation of foams with improved thermal insulating properties, reduced water absorption, and increased microbiological resistance. Due to the lower cross-linking density of bio-based PUR foams, the mechanical properties deteriorated. Uram et al. [68] developed the synthesis of bio-polyols based on rapeseed oil using epoxidation and oxirane ring opening with 1-hexanol and 1,6-hexanediol. The authors reported that the replacement of petrochemical polyol with bio-based polyol affected the physical and mechanical properties of the resulting materials due to some changes in the reactivity of the PUR system. According to the presented results, as the bio-polyol content increased, the apparent density and mechanical properties of the PUR foams decreased. Kurańska et al. [69] showed that it is possible to obtain PUR foams with sufficient thermal-insulating properties by substitution of the petrochemical polyol with rapeseed oil-based bio-polyol at an amount up to 30 wt.%. The replacement of the petrochemical polyol with a bio-polyol reduced the reactivity of the reaction mixture, leading to the formation of foams with smaller cells and lower apparent density. Authors reported that foams containing 30 wt.% of bio-based polyol exhibited slightly worse, but still satisfactory, mechanical properties. Kairytė et al. [70] synthesized rapeseed oil-based polyols modified with propylene glycol and glycerin. It was found that the application of bio-polyol modified with glycerol resulted in the formation of PUR foams with increased cross-linking density and improved thermal-insulating properties.

#### 5.1.3. Castor Oil-Based Polyols

Ogunniyi [71] developed the synthesis of castor oil-based polyols for the production of thermal-insulation materials. Such developed bio-based PUR foams were characterized by satisfactory mechanical and thermal properties; however, due to the low content of OH groups and low reactivity of the PUR system, the modified materials exhibited some drawbacks, such as a long processing time and significant shrinkage of the final materials. Interesting results were presented in the work of Zhang et al. [72], who grafted diethyl phosphate onto castor oil-based polyols, improving the flame retardancy of the bio-polyols. Subsequently, the bio-based polyols were used for the production of PUR foams with improved mechanical and thermal properties, as well as better thermal-insulation properties (0.021–0.023 W·m^−1^·K^−1^). Ionescu et al. [73] modified castor oil by means of the thiol Michael addition reaction. PUR foams based on bio-polyols were characterized by a high content of closed cells, reduced apparent density, and improved thermal-insulation properties. In the work of Carriço et al. [74], PUR foams were synthesized using a renewable polyol from the simple physical mixture of castor oil and crude glycerol. PUR rigid foams developed in this way presented properties indicating great potential for use as thermal insulation materials: low apparent density (23–41 kg·m^−3^), reduced thermal conductivity (0.0128–0.0207 W·m^−1^·K^−1^), and satisfactory mechanical properties (compressive strength of 45–188 kPa and Young’s modulus of 3–28 kPa). In another work, Hejna et al. [75] developed the synthesis of bio-polyol via the condensation of polyglycerol with castor oil. Rigid PUR foams were produced at partial replacement of petrochemical polyol with 0–70 wt.% of synthesized bio-polyol. The authors reported that the substation of the petrochemical polyol with bio-polyol only at an amount of 70 wt.% reduced the cell size and deteriorated the thermal conductivity of PUR foams. The compressive strength of bio-based foams was more than 90% higher than for foams based on petrochemical polyol. Furthermore, the incorporation of bio-polyol improved the flame retardancy and thermal stability of the final materials.

#### 5.1.4. Palm Oil-Based Polyols

Arniza et al. [76] developed the synthesis of palm-based polyol for the production of low-density PUR foams via a three-step method—(i) transesterification of palm oil, (ii) epoxidation, and (iii) opening of the epoxide ring. It was observed that replacing petroleum-derived polyol with transesterified palm-derived polyol at 30, 40, and 50 wt.% reduced the apparent density and mechanical properties of PUR foams but improved their thermal insulation properties. Low-density rigid PUR foams based on palm oil-based polyol were also investigated in the study of Srihanum et al. [77]. It was found that the substitution of petroleum-based polyols by palm oil-based polyol with a hydroxyl value of 360 mgKOH·g^−1^ up to 30 wt.% increased the mechanical strength of the final foams; however, a further increase in the amount of biopolymer led to a deterioration of the mechanical and insulation properties. Among the all samples, the best insulating properties were observed for PUR foams containing 10 wt.% of palm oil. Marcovich et al. [78] prepared polyurethane foams using a 70 wt.% palm oil-based bio-polyol with a petrochemical polyether polyol. The polyol mixture used was characterized by high viscosity; therefore, the mixture was heated before mixing with the isocyanate. However, the foaming reaction was still characterized by a low rate due to the increase in bio-polyol content. The samples had better thermal conductivity compared to the reference foams. In addition, the modified foams retained good dimensional stability.

Jaratrotkamjorn et al. [79] also worked on the development of a polyurethane foam from palm soy oil and also from a natural rubber-based bio-polyol. The production process involved an epoxidation reaction of the palm-oil double bonds, followed by a complete opening of the oxirane ring. Natural rubber, on the other hand, was subjected to oxidative degradation using periiodic acid and sodium borohydride. The process is the first example of the use of natural rubber in a mixture with a palm oil-based bio-polyol. The samples exhibited improved hardness; in addition, the presence of the rubber resulted in increased the compressive strength.

#### 5.1.5. Polyols Based on Various Compounds

Paciorek-Sadowska et al. [80] synthesized a new type of oil from *Oenothera biennis* seeds for the preparation of PUR foams with various content levels of the bio-polyol. The study showed that the addition of bio-polyols resulted in the formation of PUR foams with well-defined cellular structures and a high content of closed cells. A slight improvement in the thermal insulation properties and aging resistance was also observed. Polyether polyols for the production of rigid PUR foams were synthesized in a two-step method via the polycondensation of pure glycerol and a further propoxylation process in the work of Ionescu and Petrovic [81]. PUR foams based on bio-polyols showed improved physical and mechanical performances, e.g., low friability and good dimensional stability under critical conditions. Luo et al. [82] reported on the development of the synthesis of bio-polyols from crude glycerol, with a hydroxyl number of 480 mgKOH·g^−1^ and viscosity of about 25 Pas. PURs based on bio-polyols showed an apparent density and mechanical strength comparable to their petroleum-based analogs. Paruzel et al. developed bio-based polyols via the transesterification of coconut oil [83]. The resulting bio-polyols were used for the production of low-density PUR foams with an apparent density of 40–44 kg·m^−3^, high content of closed cells (>91%), and suitable thermal conductivity (0.0023 W·m^−1^·K^−1^). Rigid PURs were also synthesized from microalgae-based polyols. Microalgae from different sources were converted into bio-polyol via different routes, such as (i) epoxidation and opening of ring [84,85], (ii) hydroformylation [86], (iii) liquefaction in crude glycerol [87], or (iv) ozonolysis and reduction [88]. Despite of the applied method, all bio-polyols possessed the hydroxyl number above 300 mgKOH·g^−1^ and physicomechanical properties comparable to petroleum-based polyols. In all cases, the final foams were obtained by the incorporation of glycerol into the PUR system. PUR foams based on 25 wt.% of microalgae-based polyol synthesized without the oxirane opening process were first reported in the work of Arbenz et al. [84]. The authors Reference [89] carried out a comparative study of foams obtained from polyols based on different types of oil. The first and second polyols were produced via transesterification and transamidation, while the third polyol was produced via epoxidation and ring-opening reactions. In the next step, foam samples were made on the basis of the presented substrates and compared with foams made on the basis of petrochemical products. The foams based on environmentally friendly ingredients had comparable densities of around 36–38 kg/m^3^. The value of the thermal conductivity coefficient increased with the ageing time carried out over a period of 24 h, 7 days, 30 days, 3 months, and 3 years. The weakest thermal conductivity results were for foams obtained from bio-polyols made by the transamidation reaction. In contrast, the results of thermal stability and flammability tests were more favorable for samples modified with the products of the transamidation and transesterification reaction of rapeseed oil. Paciorek-Sadowska et al. [90] worked on the preparation of a new bio-polyol based on Oenothera biennis seed oil and 2,2-mercaptodiethanol (2,2-MDE). The process to obtain the polyol was carried out in two steps. Initially, the oxidation of the double bond of unsaturated fatty acid residues was carried out, followed by epoxide ring-opening reactions. The modified foams showed improved properties compared to the reference samples. In Reference [91], polyurethane foam was developed from a bio-polyol made from rapeseed straw and cellulose nanocrystals (CNCs). In this work, 40% bio-polyol and 1–6% cellulose were used as modifiers. The foam containing 20% bio-polyol showed the best physicochemical properties; a higher content resulted in a drastic decrease in properties. The use of CNC at 4% improved the Young’s modulus by 590%, and the compressive stress increased by 150%.

### 5.2. Lignin-Based Polyol

Other renewable sources used for the production of bio-polyols to develop environmentally friendly PUR products are lignocellulosic materials [92]. Cellulose is an attractive material for industry because, unlike vegetable oils, it does not compete with food resources [93]. The conversion of lignocellulosic biomass into the mixture of aliphatic and aromatic bio-polyols is performed in the solvolysis liquefaction. In general, solvolysis liquefaction can be performed via two different routes: (I) in the presence of phenol or (II) with polyhydric alcohols. Solvolysis liquefaction of biomass in the presence of phenol results in bio-polyols reach in phenols, which can be used for the synthesis of phenolic resins. Bio-polyols with low concentrations of phenolic products are synthesized in solvolysis liquefaction with polyhydric alcohols. Such developed bio-polyols are dedicated to the production of PUR foams. Typical lignocellulosic biomasses, such as cellulose derivatives [94], lignin derivatives [95], straws [96], barks [97], bagasse [98], wood [99], apricot stone shells [100], jute fibers [101], cork [102], cotton [103], and sawdust [104], have been liquefied by appropriate solvents (various glycols) to produce bio-based polyols for PUR foams.

Previous results have demonstrated that replacing, at least partially, the typical petroleum-based polyol with this type of material afforded foams with comparable foaming kinetics, density, cellular morphology, and thermal conductivity. According to the results presented by Li et al. [105], the incorporation of liquefied polyol reduced the flammability of the foams due to the aromaticity of the lignocellulosic biomass. PUR foams synthesized from lignin-based polyol exhibited better mechanical strength and improved thermal stability. Similar results were reported in the work of Arbenz et al. [106] in the case of PUR foams modified with 25, 50, 75, and 100 wt.% of tannin oil-based polyol. Compared to PUR foams modified with 25, 50, and 75 wt.% of tannin-based polyol, the best mechanical performance was observed for PUR foams based on 100 wt.% of tannin-based polyol. It was found that this could be related to the aromatic structure of the tannin-based polyol, which contributed to the increased cross-linking density of the final materials. Kosmela et al. [107] liquefied the cellulose in crude glycerol and reported that such developed bio-polyols can be successfully used for the production of PUR foams with properties comparable with petroleum-based foams. It was found that the replacement of the petroleum polyol by bio-polyol (up to 70 wt.%) increased the apparent density and affected the morphology of PUR foams, leading to the formation of larger cells. The incorporation of bio-polyol did not affect the mechanical performance of foams—a slight increase in mechanical strength was observed for foams containing 35 wt.% of bio-polyol. In yet another approach, bio-polyols have been obtained via the acid liquefaction of waste coffee grounds [108]. The thermogravimetric analysis confirmed that bio-based PUR foams were thermally stable up to 190 °C. Similar results were obtained by Domingos et al. [109]. The authors synthesized PUR foams based on liquefied eucalyptus branches. It was confirmed that the replacement of petrochemical polyol with bio-based polyol can be an effective way to obtain PUR materials with comparable thermal properties, although with slightly deteriorated mechanical properties. Marine biomass-derived polyols were prepared by Kosmela et al. [110]. The replacement of petroleum-derived polyol with up to 30 wt.% of bio-polyol increased the reactivity of the polyol system. Due to this, the obtained PUR foams exhibited improved mechanical, thermal, and insulating characteristics when compared with synthetic PUR foams. Bio-polyol obtained from cashew-nut shells was successfully for the synthesis of rigid PUR foams with good mechanical, thermal, and fire properties by Ionescu et al. [111]. Similar results were reported by Gandhi et al. [112] in the case of foams synthesized from a bio-polyol obtained from liquefied cashew-nut shells. In the work of De Luca Bossa et al. [113], sustainable PUR foams were successfully produced starting from cardanol-based polyol and additionally enhanced with various natural fillers—walnut shells, cellulose, and diatomite. The results confirmed that the introduction of walnut shells and cellulose improved the thermal stability and mechanical properties due to the increased cross-linking density of the foam. Zhang et al. [114] reported that about 25 wt.% replacement of commercially used petroleum polyol with liquefied rice straw, wheat straw, oilseed rape straw, or corn straw polyol resulted in the formation of PUR foams with improved thermal conductivity as well as comparable mechanical performances. Similar results were reported by Xuefeng et al. [115] in the case of PUR foams synthesized from oxyalkylated kraft lignin—the replacement of petroleum-based polyol with bio-polyol improved the mechanical performances of final foams. Duval et al. [116] developed a synthesis of highly reactive lignin-based liquid polyol in the reaction of organosolv lignin with ethylene carbonate. PUR foams synthesized with 25 wt.% replacement of petroleum-based polyol exhibited properties comparable to the commercial PUR foams, e.g., closed cell content of 90% and thermal conductivity of 0.025 W·m^−1^·K^−1^. In the work of Hatakeyama et al. [117], rigid PUR foams were synthesized from lignin/molasses-based polyols at different NCO/OH ratios. It was confirmed that, depending on the mixing ratio of lignin and molasses, PUR foams can be successfully used as thermal insulation materials, with thermal conductivity ranging from 0.030 to 0.040 W·m^−1^·K^−1^. The impact of the [NCO]/[OH] ratio on the cellular structure and physicomechanical properties of bio-based PUR foams was also investigated in the work of Gao et al. [118]. Rigid PUR foams were synthesized from liquefied bamboo residue at different [NCO]/[OH] ratios. It was found that as the bamboo-based polyol content increased, the overall structure of the PUR foams became less uniform, resulting in a deterioration of their mechanical properties. In yet another study, rigid PUR foams were successfully produced through the replacement of petroleum-based polyol with 25–70 wt.% of hardwood ethanol organosolv lignin (HEL) and hardwood kraft lignin (HKL). Due to the better miscibility of HEL with petroleum-based polyol, such developed foams showed better physical, mechanical and thermal performances comparing to their HKL-based analogs. Hu et al. [119] investigated two-step liquefaction of biomass using crude glycerol as a solvent. Bio-polyol with a hydroxyl number in the range of 536–936 mgKOH·g^−1^ and dynamic viscosity of 20.6–28.0 Pas was used for the production of PUR foams. It was found that produced PUR foams exhibited better mechanical and thermal properties when compared with their analogs containing bio-polyol from a one-step biomass liquefaction. Li et al. [120] synthesized rigid polyurethane foams from fractionated lignin with high functionality and low molar mass. The process involved ethanol–organic fractionation using corn straw to precipitate the lignin. The authors characterized the respective lignin fractions showing differences in thermomechanical properties between them. In particular, lignin precipitated in ethanol at 30% or 15% was characterized by a lower molecular weight and higher active hydroxyl content, leading to foams with improved compressive strength (0.78 ± 0.02 MPa), lower thermal conductivity (0.037 ± 0.01 W m^−1^ K^−1^), and better thermal stability. The authors of another publication [121] developed an oil sorbent derived from polyurethane foam containing up to 40 wt.% hydrophobized lignin particles; it was acetylated to increase its matrix compatibility and sorption capacity.

### 5.3. Rigid PUR Foams Reinforced with Natural Fillers

**Figure 7:**
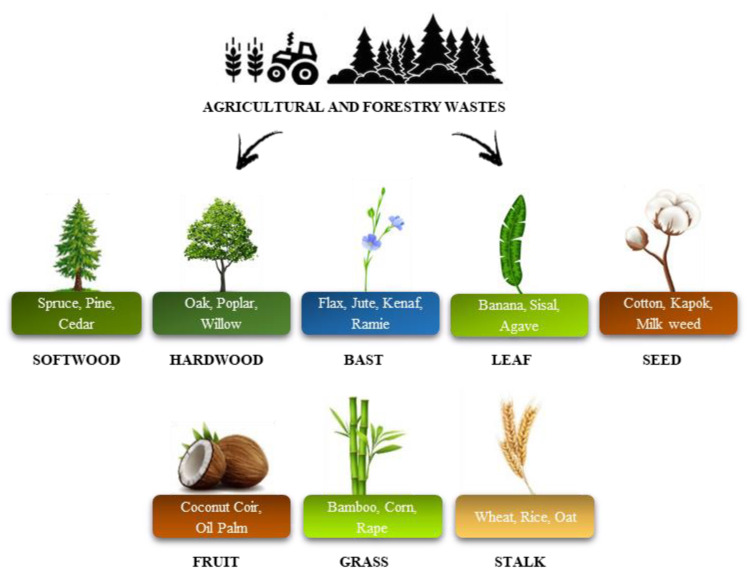
Bio-based waste from agricultural and forestry industries used as reinforcing fillers in PUR materials.

Another approach for the production of environmentally friendly PUR foams under the circular economy is the reinforcement of polymeric materials with bio-based fillers derived from forestry or agricultural wastes [122,123]. Previous studies have shown that the introduction of bio-based fillers into PUR foams improves their physical and mechanical properties, as well as provides reactive groups that can react with isocyanates. A variety of fillers derived from agricultural and forestry wastes have been studied in the literature (Figure 7).

For example, Zhou et al. [124] reported on the synthesis of semi-rigid PUR foams based on palm-oil polyol and reinforced with cellulose nanocrystals. Compared to the unmodified foams, PUR composites with improved compressive strength, superior dimensional stability in both freezing and heating conditions, and greater water uptake were obtained. With the increasing content of cellulose, the rigidity of PUR foams increased, leading to reduced deformation resilience. In another study by Zhou et al. [125], castor oil-based rigid PUR foams were reinforced with 0.25, 0.5, and 1 wt.% of carrot nanofibers. It was found that the introduction of 0.5 wt.% natural filler resulted in PUR composites with better mechanical properties, while the property decreased when the filler concentration increased to 0.1 and 1 wt.%. A similar dependence between filler concentration and mechanical properties of foams was reported by Xue et al. [126]. The authors examined PUR foams reinforced with 1, 2, and 5 wt.% of pulp fiber. It was found that the addition of bio-based filler affected the cellular morphology of foams, leading to the formation of composites with irregular and large cells. As the pulp-fiber content increased, the apparent density and compressive strength decreased. Riberio da Silva et al. [127] also reported a similar observation to that of Xue et al. When compared to the unfilled foams, the incorporation of rice-husk ash at the concertation of 1, 2, 3, and 5 wt.% decreased the mechanical properties of PUR composites due to the increased viscosity and reduced reactivity of the reaction mixtures containing bio-filler. Paberza et al. [128] prepared PUR foams based on tall-oil amide and reinforced with 0–6.3 wt.% of wheat-straw lignin. The maximum compressive strength value was shown by foams with the addition of 1.2 wt.% of lignin. A further increase in the concentration of lignin worsened the mechanical and thermal insulation properties of the composites due to the high viscosity of the reaction system and reduced efficiency of component mixing. Zieleniewska et al. [129] synthesized PUR foams with eggshells as a natural filler. The results of infrared absorption spectra confirmed that the addition of eggshell filler increased the degree of phase separation in PUR composites. PUR composites containing natural filler exhibited increased apparent density and improved compressive strength in the orientation parallel to the foam growth direction, as well as reduced water absorption, low friability, and high dimensional stability in aqueous media. A different tendency was reported by Silva et al. in the case of PUR foams reinforced with cellulose fiber residue [130]. The addition of 1–8 wt.% of bio-filler resulted in increased apparent density and decreased mechanical strength. Upon the addition of 8–16 wt.% of the filler, the apparent density increased; however, the compressive strength was decreased. Furthermore, the addition of 16 wt.% of cellulose fiber decreased the thermal conductivity, while the thermal stability remained unchanged. Interesting results were presented in the work of Prociak et al. [131], who prepared rigid PUR foams reinforced with different natural fillers, such as ground walnut shells and ultrafine cellulose at the concentrations of 1, 3, and 6 wt.%. It was found that, compared with the reference PUR foams, the reinforcement of composites with 9 wt.% of bio-fillers improved their mechanical properties. In the work of Mosiwiecki et al. [132] rigid PUR foams were reinforced with 5, 10, and 15 wt.% of wood flour. It was found that the chemical reaction between wood flour and isocyanate affected the reactivity of PUR composites and their further physicomechanical properties. As the concentration of wood flour increased to 15 wt.%, the thermal conductivity of the foams increased, but their mechanical properties decreased. Paciorek-Sadowska et al. [133] investigated PUR foams reinforced with solid parts of wheat slops in the amount of 5–30 wt.%. When compared to the reference foam, the incorporation of wheat slops at each amount helped to reduce the brittleness of PUR composites; however, the mechanical properties slightly deteriorated. Bagasse fibers were used as a reinforcing filler for rigid PUR foams in the work of Qiu et al. [134]. It was found that, with the increasing content of bagasse fibers, the mechanical performances of PUR composites decreased; however, the thermal conductivity remained almost unchanged. The incorporation of natural filler improved the dimensional stability of composites—the best stability was observed for composites containing 40 wt.% of filler. De Avila Delucis et al. [135] examined the physicomechanical properties of PUR foams reinforced with different amounts (1, 5, and 10 wt.%) of forestry-derived fillers, such as wood, bark, cones, and needles from pine trees, kraft lignin, and recycled paper sludge. The incorporation of bio-fillers resulted in the formation of PUR composites with a regular cell structure and high content of closed cells. Among the tested composites, the most promising formulations, with improved hygroscopic and mechanical properties, were reported for composites reinforced with 1 and 5 wt.% of wood. The authors reported that this may be connected with higher compatibility of wood with PUR systems, as indicated by wet chemical analyses and micrographs’ results. Rigid PUR foams reinforced with different weight ratios (1, 3, and 5 wt.%) of cellulose fibers from pineapple were prepared by Jabber et al. [136]. Cellulose fibers were chemically treated to remove hemicellulose, lignin, and other impurities. According to the results presented by the authors, the chemical treatment of the fibers reduced the cross-link ability between the cellulose fibers and PUR matrix, leading to the deterioration of the mechanical performance of composites. In the work of Uram et al. [137], rigid PUR foams were reinforced with 3, 6, 9, and 20 wt.% of biochar. When compared to the unfilled foam, the filler-containing composites exhibited reduced apparent density, deteriorated mechanical performances, and improved thermal stability. The introduction of bio-filler did not affect the thermal conductivity, which remained at the level of 0.025 W·m^−1^·K^−1^. In another work by Uram et al. [138], rigid PUR foams were reinforced with 1, 2, and 3 wt.% of cellulose filler. The incorporation of the natural filler affected the cellular structure of composites, leading to the formation of composites with a uniform structure and high content of closed cells (>90%). All composites were characterized by good thermal insulation properties with a thermal conductivity of ~0.025 W·m^−1^·K^−1^. Kuźnia et al. investigated the impact of fly ash addition on the physicomechanical properties of rigid PUR foams [139]. It was found that the addition of 5 and 10 wt.% of fly ash significantly improved the mechanical and thermal performances of PUR composites; however, the use of the filler in the amount greater than 10 wt.% deteriorated the majority of the tested properties. Paciorek-Sadowska et al. [140] developed rigid PUR foam composites, reinforced with 5–35 wt.% of grain fraction of fly ashes. When compared with unfilled foam, the produced composites exhibited reduced brittleness and higher apparent density, which contributed to the improved mechanical performances of the developed composites. In another work, Liszkowska [141] evaluated the properties of rigid PUR foams reinforced with 2.5–15 wt.% of ground coffee. It was found that the addition of natural filler shortened the processing times and affected the cellular structure of PUR composites, resulting in the formation of a more irregular structure with slight defects, which were caused by the presence of coffee particles. With the increasing content of coffee filler, the apparent density increased, and the mechanical properties slightly deteriorated. Compared to the reference foam, the reinforced composites were characterized by increased impregnability and absorptivity. The incorporation of the filler did not affect the aging parameters of modified foams. Interesting results were reported in the work of Wrześniewska-Tosik et al. [142]. Rigid PUR foams were reinforced with 10 wt.% of keratin fibers, additionally modified with selected flame-retardant compounds, such as Fyrol (phosphate ester), expandable graphite, aluminum hydroxide, magnesium hydroxide, zinc oxide, and ammonium polyphosphate. It was found that the incorporation of keratin fillers significantly reduced the amount of smoke generated during the burning process. The incorporation of the filler increased the ignition time and decreased the maximum heat release rate. The impact of different amounts of microcellulose (3, 6, and 9 wt.%) on the physicomechanical properties of PUR foams synthesized from rapeseed oil-based polyol was also examined by Kurańska et al. [143]. It was noted that, with the addition of natural filler, the flammability of modified foams increased significantly due to the presence of microcellulose, which is known as a highly flammable material. It was found that the addition of the microcellulose significantly increased the viscosity of PUR systems, decreasing their reactivity. The addition of microcellulose at each amount did not affect the apparent density of composites; however, the filler incorporation had a positive effect on the mechanical performances of the modified composites. A similar dependence between apparent density and mechanical performance of reinforced composites was reported in the work of Luo et al. [144]. The authors used a microcrystalline cellulose as a reinforcement for soy-based PUR foams. It was found that the addition of 1, 5, and 10 wt.% of microcellulose increased the apparent density of PUR foams. The improvement of the mechanical performance was observed for composites reinforced with 1 wt.% of the filler. Soy protein in various concentrations (2.4, 4.8, 7.2, and 9.6 wt.%) was used as a reinforcement for rigid PUR foams in the work of Tian et al. [145]. It was reported that strong bonding interaction occurred between functional groups of soy protein and PUR matrix, increasing the cross-linking density of PUR composites. The addition of soy protein increased the apparent density of composites and improved their mechanical performance. Due to the cross-linking effect, the thermal stability of PUR foams containing soy protein particles was enhanced. Khaleel et al. [146] investigated the impact of the addition of different content (3, 6, 9, 12, and 15 wt.%) of turkey-feather fibers on the physicomechanical properties of PUR foams. It was found that the addition of feather fibers at a low concentration improved the thermal stability of composites; however, this improvement was lost at higher concentrations of the filler. Among all tested composites, the most preferred properties were noted for samples containing 3 wt.% of keratin fibers. In comparison with unfilled foams, those samples were characterized by a better thermal insulating performance (lower value of thermal conductivity) and lower air permeability. Beyond 3 wt.% of filler, some deterioration in thermal and mechanical performances has been observed. The authors concluded that this may be connected with the fact that, at lower filler concentrations, the samples were characterized by uniform cell distribution, while an inhomogeneous cellular structure with an accumulation of filler particles was observed at a higher loading of the filler. The research of Sair et al. [147] confirmed that the addition of 5, 10, 15, 20, and 25 wt.% of hemp fibers provides a promising attempt for the production of thermal insulating materials. The mechanical properties, such as mechanical and flexural strength, increased up to 15 wt.% of natural filler. Above 20 wt.% of filler, the mechanical properties decreased due to agglomeration of filler particles and inhomogeneity of the composite structure. Interesting results were presented in the work of Delcius et al. [148] in the case of rigid PUR foams reinforced with different types of forestry wastes, such as bark, wood, kraft lignin, and paper sludge. It was shown that the addition of pine bark is preferable for the use in rigid PUR foams, which require a great ultraviolet resistance. On the contrary, the incorporation of wood chips resulted in the formation of composites with reduced apparent density and greater photo-degradation effects when compared with the dark ones. Outshabi et al. [149] reinforced rigid PUR foams with different contents (5, 10, and 20 wt.%) of date-palm particles. The study showed that the thermal and mechanical properties of composites reinforced with 5 and 10 wt.% of the filler were comparable to those of commercial composites, but as the content of date-palm particles increased to 20 wt.%, the apparent density of the composites increased, leading to a deterioration in thermal-insulation properties. The results of scanning electron microscopy confirmed that the introduction of 20 wt.% filler caused a deterioration of the mechanical properties of the composites due to the agglomeration of filler particles in the reaction mixture and, consequently, a disturbed foaming process. Husainie et al. [150] investigated the impact of the addition of 1, 2, and 5 wt.% of different fillers, such as hazelnut, cellulose, chitin, and eggshells, on the mechanical and thermal performances of PUR foams. Among all the samples, the addition of 1 wt.% of natural fillers resulted in the formation of PUR composites with improved mechanical and resiliency properties. In the work of Kairytė et al. [41], rigid PUR foams were reinforced with 5, 10, 15, and 20 wt.% of paper-waste sludge (PWS) particles. It was confirmed that the most preferably filler particle content is 5 wt.%. Compared with the unfilled foams, the addition of 5 wt.% of the filler resulted in the formation of PUR composites with improved mechanical strength, lower thermal conductivity, and reduced water-vapor resistance. With increasing filler content, the thermal-insulating properties and mechanical strength decreased. In another study by Kairytė et al. [151], rigid PUR foams were reinforced with various amounts (10, 20, 30, 40, and 50 wt.%) of bottom-waste ash. It was found that bottom waste-ash particles may act as a plasticizer in PUR foams, increasing the dynamic viscosity of modified systems and reducing the reactivity of the systems, and these results were confirmed by a reduced foaming temperature. The addition of filler affected the cellular structure of PUR composites—up to 10 wt.% filler; the composites were characterized by a uniform structure with a regular shape cells, but further addition of filler led to composites with an irregular structure, with visible loose particles located in the cell windows. Compared with unfilled foams, the addition of filler in the amount of 10–40 wt.% improved the thermal and mechanical properties of the composites, but the addition of 50 wt.% of the filler was found to be an excessive amount, leading to a deterioration of the aforementioned properties. A few years ago, Husainie et al. [152] conducted a study using fillers in the form of lignin, chitin, chitosan, hazelnut shell, and polysaccharide to determine their effect on the properties of polyurethane foams. For this purpose, the researchers made foam samples using 1, 2.5, and 5% modifier. They carried out morphological tests and showed that chitin and hazelnuts showed good dispersion in the foams; in addition, they improved the tensile strength and increased the elasticity, while reducing the hardness of the polyurethane foam. The polysaccharide filler showed similar improvements in tensile performance, while lignin and chitosan reduced the mechanical properties of the foam, indicating their low compatibility with polyurethane foam. In Reference [153], rigid PUR foams were reinforced with 10, 20, and 30 wt.% of sunflower press cake (SFP) and sunflower press cake impregnated with liquid glass (LG-SFP). It was found that both kinds of fillers affected the rheology and foaming behavior of polyol premixes, affecting the further morphological, mechanical, and thermal properties of reinforced composites. A morphological analysis revealed that the incorporation of each amount of filler resulted in the formation of dimensionally stable composites with a great number of closed cells. Besides the increased dynamic viscosity and apparent density, the reinforced composites were characterized by improved mechanical performances and reduced thermal conductivity. Compared with unfilled foam, the water absorption was reduced almost two times. The improvement in thermal properties was noted only for samples reinforced with LG-SFP. No improvement was observed in the case of composites reinforced with SFP. Głowacz-Czerwonka et al. [154] have applied a modifier in the form of sunflower husks, rice husks, and buckwheat hulls at various weight ratios (5 wt.%, 10 wt.%, and 15 wt.%). The authors investigated the effects of bio-fillers on the structure, mechanical, and thermal properties, as well as the dimensional stability, water absorption, and apparent density of RPUF. The results confirm that the foam modified with this type of filler, compared to the reference sample (no filler), has a higher apparent density (38–51 $\frac{kg}{m^{3}}$), lower water absorption (18–32%), and higher compressive strength (50–90 kPa). In addition, the flammability of the samples was tested using a cone calorimeter. The foam containing 5 wt.% rice husks showed the highest fire resistance. The results obtained indicate the possibility of introducing bio-fillers into polyurethane materials as ingredients that can improve the properties of these materials. Anwar et al. [155] focused on the development of polyurethane foams with reduced water absorption, which is limited due to the hydrophobic nature of polyurethane foams. This type of material could find a wide range of applications in plant cultivation, among others. A good way to improve this property is to use pulp, which is a waste material. The authors conducted a study, according to which it was found that water absorption and water retention are correlated with cell structure and improve with increasing cell size. It was found that polyurethane foam modified with 4 wt.% pulp showed satisfactory water-absorption (11.5%) and water-retention (75.9%) properties among the samples. The use of pulp represents a good use of pulp in the context of waste management. Additionally, all the summarized properties of the researches analyzed are presented in Table 1.

### 5.4. Use of Paper Waste in the Production of Polyurethane Foams

Kairyte et al. [41] also worked on the use of paper waste as a modifier for polyurethane foams. The authors ground the paper into a powder with a grain size smaller than 0.063 mm in order to achieve a better specific surface area of the modifier. The results obtained suggest that the best amount of filler in polyurethane foam is 5% by weight of the foam. The modifier improves the thermal conductivity, water vapor resistance, density, compressive strength, and modulus of elasticity compared to a sight sample that was not modified with paper.

Moreover Kairytė et al. [42] research demonstrates the problem of dimensional instability in foams. The use of modifiers in the form of paper waste can lead to collapse of the structure of polyurethane foams. For this purpose, the authors use propylene glycol of biological origin. The results obtained confirmed that this raw material allows for the development of dimensionally and structurally stable polyurethane foams used as a thermal insulation layer with the following properties: density in the range of 40–50 kg/m^3^, compressive strength in the range of 193–243 kPa, thermal conductivity in the range of 0.0349–0.0359 W/(m-K), long-term water absorption in the range of 6–11 vol.% and a water vapor transmission rate in the range of 26.2–40.9. The additive has a positive effect on polyurethane foams. The above-cited publications confirm the possibility of using paper waste as a filler for polyurethane foams.

### 5.5. Improving the Sound Insulation Properties of Polyurethane Foams

Polyurethane foams have versatile performance properties used particularly in the construction industry, which is looking for new solutions. PU foams have a low sound-absorption capacity due to their closed pore structure. Therefore, Chanlert et al. [156] developed compositions for polyurethane foam containing a non-reactive filler that can prevent the formation of closed cells in the process. The researchers prepared a rigid polyurethane foam prepared from polyether polyol and polymeric methylene diisocyanate (p-MDI), with water as blowing agent. Natural rubberwood sawdust was used as filler. The percentage of rubberwood sawdust in the polyurethane foam composites varied by 0, 1, 3, 5, and 7%. Measurements of the sound absorption coefficient were made using the tube impregnation technique. The results showed that closed-cell rigid polyurethane foam, which is a reference sample and contains no filler, exhibits poor acoustic absorption. In addition, the results of the sound-absorption coefficient showed that the sample with a higher amount of rubberwood sawdust allowed the sound wave to be absorbed over a wider frequency range. The authors of another study [157] investigated the possibility of using natural fibers derived from artichoke stems. The material was subjected to surface modification with sodium hydroxide solution at concentrations of (5 and 10%) for (5, 10 and 15 min). The resulting material was introduced into the polyurethane foam in the following proportions (5, 10, 15, and 20%). Tests to determine the mechanical properties of the composites, such as flexural strength and elongation, were carried out for each of the prepared samples. The samples with the best properties were qualified for thermal and acoustic conductivity tests, and their morphological properties were examined. It was found that waste fibers from artichoke stalks could provide good mechanical, thermal, and acoustic properties as modifiers for polyurethane foams. In Reference [158], the authors also worked on improving the sound-absorption capacity of polyurethane foams. They used rice-plant waste as a modifier. Fiber-filled foams that had been modified with 10% NaOH for 15 min were tested, and unmodified samples were also made. Filler amounts of 5%, 10%, 15%, and 20% by weight were used. It was found that modification of the fibers with the NaOH solution did not have the expected results, and that the samples filled with 5% fibers that had not been treated showed the best sound-absorption properties and had the best strength. Ekici et al. [159] also worked on improving the acoustic properties of PU foams using compounds of natural origin. The authors used tealeaf fibers and showed that this type of modification had a positive effect on sound-absorption properties. The results show a consistent increase at frequencies between 1.6 and 6.3 kHz. Sound absorption is four times higher for the modified sample compared to the unmodified sample at some frequencies. The inclusion of 8–16% modifier improves the sound-absorption values for all frequency ranges. Increasing the tea leaf fiber content of the foam matrix to 24% improves sound-absorption properties in the mid-frequency ranges.

## 6. Types and Applications of Polyurethane Materials

The broad raw material base (especially concerning polyol raw materials) means that PU can be produced in the form of numerous material groups, such as the following:

- Rigid and flexible foams,
- Thermoplastics,
- Thermosetting plastics,
- Coatings,
- Adhesives,
- Sealants,
- Elastomers.

**Figure 8:**
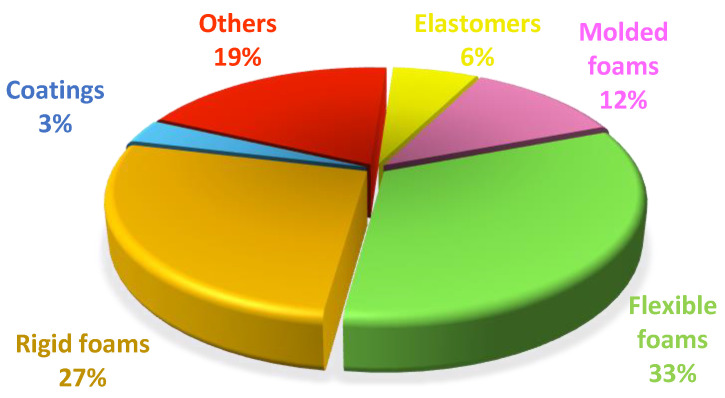
World market breakdown of polyurethanes.

The development of polyurethanes came in the 1930s by Otto Bayer and his colleagues at I.G. Farbenindustrie in Leverkusen, Germany, which found use as an alternative to rubber during the Second World War [13,16]. In the following years, there has been a rapid increase in the production and use of PU mainly in the field of polyurethane foams. The versatility of their use is due to the wide range of properties that can be obtained by modifying the composition used. As a result, they are widely used in a variety of industries (Figure 8).

The largest market for their use is the construction industry, which uses foams to produce thermal or acoustic insulation. Methods of spray-coating attics or building walls with polyurethane foam are popular, as they minimize the share of thermal bridges in such insulation. Polyurethane foams are used as filling for mattresses or as insulation in refrigerated food trucks or tankers. [10]. The literature reports the use of foams in gas absorption, water purification, piezoresistive pressure sensors, soft armor, tissue engineering scaffolds, EMI shielding or porous electrodes, and much more [29]. According to 2019 data, the foam industry accounted for more than 65% of the polyurethane market share. In contrast, the global market value as of 2023 represents USD 83.54 billion, with a projection of USD 111.16 billion by 2029, where the PU market is forecast to be worth USD 111.16 billion [160].

## 7. Recycling of Polyurethane Foams

The widespread use of polyurethane foams is associated with the problem of waste generation; they are mainly landfilled or incinerated. This solution is not correct due to the environmental pollution of the toxic compounds used in their production. The lack of a recycling process for foams also increases the cost of producing this material. Approximately 10 Mt of polyurethane is produced each year. In the 1990s, concerns were raised about their accumulation in landfills in significant quantities or combustion. Consequently, the literature presents recycling methods based on microbial or enzymatic degradation. In the following section, several recycling methods are presented, divided into physical, chemical, and biological recycling (Table 2) [161,162,163,164].

### 7.1. Chemical Recycling

Chemical recycling of polyurethane waste is a necessary process that can reduce the volume of virgin polyol production. This is difficult due to the need for significant amounts of solvents and slow reaction rates [166]. The method involves a gradual depolymerization into oligomers or smaller particles that can be used as reactants for new PU foams in the future. In the past years, there have been several publications presenting methods for the chemical recycling of foams. These processes are based on hydrolysis or aminolysis. The hydrolysis process is based on the use of superheated steam to hydrolyze the urethane bonds, resulting in polyols and amines, which can be reused in the production of polyurethanes. The aminolysis process, on the other hand, focuses on the breaking of urethane bonds with amines, resulting in oligomeric urethanes and amines. The most common method used in industry today is glycolysis, which uses high-boiling glycols to lead to the decomposition of [165,167]. Liu et al. [166] developed a rapid method (<10 min) for the acidolysis of model-based flexible polyurethane foam (TDI) at 200 °C, using maleic acid (MA) with recycled polyol recovery with an efficiency of 95%. The resulting product exhibits comparable properties to the virgin polyol. A major advantage of this process is that no solvents are required. Lignin is used in the production of polyurethane foams, which is an attractive bio-based material. The authors of this publication [168] developed a chemical recycling technique that prevents the loss of functionality of lignin and leads to a material capable of synthesizing non-isocyanate polyurethane foams. The technique focused on depolymerization of the polymer and isolation of the lignin with increased solubility and hydroxyl content.

### 7.2. Physical and Mechanical Recycling

This method involves mechanically turning the foams into powder, which can then be reused in PU production. Various methods have been developed to produce recycled foams. The most common ones boil down to hot pressing the foam powder with an adhesive. The process involves grinding the waste foams, and then they are coated with glue and melted in a water jet, at a temperature of about 100 °C, and in a subsequent step, pressed usually at a pressure of 30 to 200 bar. The water vapor reacts with the prepolymer or binder, leading to the re-bonding of the polyurethane. The resulting product can be reused as tire covers, sports mats, or car coverings. However, the properties of such a material will be inferior compared to the original properties [165,169]. Researchers are also focusing on developing a method to use waste polyurethane foams as fillers in other materials. Gomez-Rojo et al. [170] publish the results of a study that describes the use of polyurethane foams in building materials. The authors confirmed the possibility of using these wastes in gypsum matrices for plastering walls. The results confirmed that the foams remain inert and do not leach when applied to the wall.

### 7.3. Biological Recycling

The process of recycling polyurethane foams can involve the use of living microorganisms. Fungi and bacteria, under aerobic or anaerobic conditions, react with the macromolecular chains to shorten them, causing a significant decrease in the molecular weight of the polymer. Biodegradation proceeds in three successive stages: disruption of the macromolecular chains and formation of oligomeric structures, further degradation to small particles, and then final conversion to CO_2_ and H_2_O under aerobic conditions and CH_4_ under anaerobic conditions [171]. Magnin et al. developed a method combining biological and chemical processes involving the enzymatic degradation of polyurethane foam from which degradation products were obtained that could be used in other chemical processes. The researchers degraded foam obtained from polycaprolactone and toluene diisocyanate. The degradation process resulted in a weight loss of 25% in 24 h [172]. Biological degradation is more environmentally friendly than thermochemical degradation [165]. The author’s research [173] showed that mealworm (Tenebrio Molitor) larvae effectively chew and ingest polyurethane foams, resulting in a significant weight loss of 67% after 35 days. The study revealed that polyurethane is converted to low-molecular-weight oligomers by these organisms. The authors of the cited publication [174] showed that polyurethane foams can be biodegraded by marine myco-organisms in ex situ aquatic environments. SEM, FTIR, and gas chromatography–mass spectrometry (GCMS) analysis confirmed that the microorganisms led to the depolymerization of PU into constitutive diols, acids, and other PU molecular fragments. The fact that the foams were biodegraded in the natural environment and their degradation products were absorbed into the biomass is a positive aspect.

## 8. Summary and Outlook

Polyurethane foams are of great interest to many industries due to their wide distribution of properties. They have low density, low thermal conductivity, and good mechanical properties, making it possible to use them as thermal and acoustic insulators. They are also used as construction materials or as mattress fillings. However, they face problems, such as the toxicity of the compounds they are made of, or their flammability and release of harmful gases during the combustion process. Therefore, research is required to develop substitute compounds from renewable sources. A promising issue is the use of plant-derived polyols due to the high availability, low cost, and renewable nature of vegetable oils. In recent years, many scientific publications have shown that polyurethane foams of plant origin can compete with conventional PUFs. Another promising issue is the use of plant-derived fillers in the PU foam industry, mainly containing cellulose compounds, which, when contained in the foam matrix, improve the properties of the foam. The development of biodegradable PU foams will contribute to the development of a sustainable environmental future and the widespread use of PU foams in various industries.

## Figures and Tables

**Table 1:** Summary table of density, thermal conductivity, and compressive-strength results for rigid polyurethane foams modified with natural filler.

Filler Type	Density, g/cm^3^	Thermal Conductivity, W/m*K	Compressive Strength, Kpa
Tung oil-based polyol/rice-husk ash filler [127]	0.09–0.05	-	3–5
Tall oil-based polyol/wheat straw-lignin filler [128]	0.045–0.06	0.0324	30–35
Cellulose fibers filler [39]	0.029–0.037	0.0220–0.0300	160–175
Castor oil-based polyol/wood flour filler [132]	0.021–0.037	0.0390	2100–3400
Wheat-slop filler [133]	0.033–0.025	0.0307	80–200
Bagasse-fiber filler [134]	0.041–0.060	0.025–0.030	180–240
Pineapple filler [136]	0.053–0.054	-	300–400
Ground-coffee filler [141]	0.046–0.054	-	150–240
Turkey feather-fiber filler [146]	0.038–0.040	0.0291	-
Sunflower press-cake filler [154]	0.066–0.086	0.0294–0.0321	160–320
Liquid glass-impregnated sunflower press-cake filler [154]	0.054–0.068	0.0319–0.0328	120–200

**Table 2:** Overview of possible recycling processes for polyurethane foams [165].

Type of Recycling	Process	Products
Mechanical recycling	Extrusion, pressing, injection molding	Products with deteriorated properties compared to the original ones
Physical recycling	Pressing with glue	Products with deteriorated properties compared to the original ones
Chemical recycling	Hydrolysis, glycolysis, aminolysis, acidolysis, phosphorolysis	Monomers, oligomers
Thermochemical recycling	Pyrolysis, gasification, hydrogenation	Chemical compounds, fuels
Biological recycling	Biodegradation	CO_2_, H_2_O, CH_4_
Energy recycling	Combustion	Energy

## Data Availability

All the data are available within the article.

## References

[1] Combating Climate Change. https://www.europarl.europa.eu/factsheets/en/sheet/72/walka-ze-zmiana-klimatu.

[2] (2010). EU Directive 2010/31/EU of the European Parliament and of the Council (on the Energy Performance of Buildings). https://eur-lex.europa.eu/LexUriServ/LexUriServ.do?uri=OJ:L:2010:153:0013:0035:EN:PDF.

[3] (2012). EU Directive 2012/27/EU of the European Parliament and of the Council. https://eur-lex.europa.eu/LexUriServ/LexUriServ.do?uri=OJ:L:2012:315:0001:0056:en:PDF.

[4] Paris Agreement on Climate Change. https://www.consilium.europa.eu/en/policies/climate-change/paris-agreement/.

[5] Serrano A., Borreguero A.M., Garrido I., Rodríguez J.F., Carmona M. (2016). Reducing Heat Loss through the Building Envelope by Using Polyurethane Foams Containing Thermoregulating Microcapsules. Appl. Therm. Eng..

[6] Kim C., Youn J.R. (2000). Environmentally Friendly Processing of Polyurethane Foam for Thermal Insulation. Polym. Plast. Technol. Eng..

[7] Vest N.A., Kolibaba T.J., Afonso A.O., Kulatilaka S.A., Iverson E.T., Grunlan J.C. (2022). Acid-Doped Biopolymer Nanocoatings for Flame-Retardant Polyurethane Foam. ACS Appl. Polym. Mater..

[8] Palen B., Kolibaba T.J., Brehm J.T., Shen R., Quan Y., Wang Q., Grunlan J.C. (2021). Clay-Filled Polyelectrolyte Complex Nanocoating for Flame-Retardant Polyurethane Foam. ACS Omega.

[9] Lin B., Yuen A.C.Y., Chen T.B.Y., Yu B., Yang W., Zhang J., Yao Y., Wu S., Wang C.H., Yeoh G.H. (2021). Experimental and Numerical Perspective on the Fire Performance of MXene/Chitosan/Phytic Acid Coated Flexible Polyurethane Foam. Sci. Rep..

[10] Rostami-Tapeh-Esmaeil E., Rodrigue D. (2023). Morphological, Mechanical and Thermal Properties of Rubber Foams: A Review Based on Recent Investigations. Materials.

[11] Brzeska J., Piotrowska-Kirschling A. (2021). A Brief Introduction to the Polyurethanes According to the Principles of Green Chemis-try. Processes.

[12] Oertel G. (1994). Polyurethane Handbook: Chemistry, Raw Materials, Processing, Application, Properties.

[13] Szycher M., Szycher M. (2012). Szycher’s Handbook of Polyurethanses.

[14] Chattopadhyay D.K., Webster D.C. (2009). Thermal Stability and Flame Retardancy of Polyurethanes. Prog. Polym. Sci..

[15] Skleničková K., Abbrent S., Halecký M., Kočí V., Beneš H. (2022). Biodegradability and Ecotoxicity of Polyurethane Foams: A Review. Crit. Rev. Environ. Sci. Technol..

[16] Bayer O. (1947). Das Di-Lsocganat-Poluadditionsverfahren (Polyurethane). Angew. Chem..

[17] Crescentini T.M., May J.C., McLean J.A., Hercules D.M. (2019). Mass Spectrometry of Polyurethanes. Polymer.

[18] Dutta A.S. (2018). Polyurethane Foam Chemistry. Recycling of Polyurethane Foams.

[19] Gogoi R., Alam M.S., Niyogi U.K. (2013). Effect of Soft Segment Chain Length on Tailoring the Properties of Isocyanate Terminated Oolyurethane Prepolymer, a Base Material for Polyurethane Bandage. Int. J. Res. Eng. Technol..

[20] Wang Z., Wang C., Gao Y., Li Z., Shang Y., Li H. (2023). Porous Thermal Insulation Polyurethane Foam Materials. Polymers.

[21] Janik H., Sienkiewicz M., Kucinska-Lipka J. (2014). Polyurethanes. Handbook of Thermoset Plastics.

[22] Akindoyo J.O., Beg M.D.H., Ghazali S., Islam M.R., Jeyaratnam N., Yuvaraj A.R. (2016). Polyurethane Types, Synthesis and Applications—A Review. RSC Adv..

[23] Kaikade D.S., Sabnis A.S. (2023). Polyurethane Foams from Vegetable Oil-Based Polyols: A Review. Polym. Bull..

[24] Sonnenschein M.F. (2014). Polyurethanes: Science, Technology, Markets, and Trends.

[25] Randall D., Lee S. (2002). The Polyurethanes Book.

[26] Javni I., Zhang W., Petrović Z.S. (2003). Effect of Different Isocyanates on the Properties of Soy-based Polyurethanes. J. Appl. Polym. Sci..

[27] Allport D.C., Gilbert D.S., Outterside S.M., Allport D.C., Gilbert D.S., Outterside S.M. (2003). MDI and TDI: Safety, Health and the Environment.

[28] Liu H., Zhao X. (2022). Thermal Conductivity Analysis of High Porosity Structures with Open and Closed Pores. Int. J. Heat Mass Transf..

[29] Zhao X., Brozena A.H., Hu L. (2021). Critical Roles of Pores and Moisture in Sustainable Nanocellulose-Based Super-Thermal Insulators. Matter.

[30] Zhao Y., Zhong F., Tekeei A., Suppes G.J. (2014). Modeling Impact of Catalyst Loading on Polyurethane Foam Polymerization. Appl. Catal. A Gen..

[31] Yasunaga K., Neff R.A., Zhang X.D., Macosko C.W. (1996). Study of Cell Opening in Flexible Polyurethane Foam. J. Cell. Plast..

[32] Dworakowska S., Bogdał D., Zaccheria F., Ravasio N. (2014). The Role of Catalysis in the Synthesis of Polyurethane Foams Based on Renewable Raw Materials. Catal. Today.

[33] Yadav A., de Souza F.M., Dawsey T., Gupta R.K. (2022). Recent Advancements in Flame-Retardant Polyurethane Foams: A Review. Ind. Eng. Chem. Res..

[34] McKenna S.T., Hull T.R. (2016). The Fire Toxicity of Polyurethane Foams. Fire Sci. Rev..

[35] Cellulose. https://pl.wikipedia.org/wiki/Celuloza.

[36] Mort R., Peters E., Griffin E., Curtzwiler G., Vorst K., Jiang S. (2023). Low-Isocyanate Polyurethane Foams with Improved Stability and Compression Modulus Prepared from Biosourced and Landfill-Diverted Materials. ACS Appl. Polym. Mater..

[37] Stanzione M., Oliviero M., Cocca M., Errico M.E., Gentile G., Avella M., Lavorgna M., Buonocore G.G., Verdolotti L. (2020). Tuning of Polyurethane Foam Mechanical and Thermal Properties Using Ball-Milled Cellulose. Carbohydr. Polym..

[38] Beaufils-Marquet M., Blanchet P., Hussain A., Landry V. (2023). Investigation of Cellulose Filaments as Filler in Rigid Insulating Polyurethane Foam. BioResources.

[39] Sture B., Vevere L., Kirpluks M., Godina D., Fridrihsone A., Cabulis U. (2021). Polyurethane Foam Composites Reinforced with Renewable Fillers for Cryogenic Insulation. Polymers.

[40] Zhang J., Hori N., Takemura A. (2021). Reinforcement of Agricultural Wastes Liquefied Polyols Based Polyurethane Foams by Agricultural Wastes Particles. J. Appl. Polym. Sci..

[41] Kairytė A., Kirpluks M., Ivdre A., Cabulis U., Vėjelis S., Balčiūnas G. (2018). Paper Waste Sludge Enhanced Eco-efficient Polyurethane Foam Composites: Physical–Mechanical Properties and Microstructure. Polym. Compos..

[42] Kairytė A., Vaitkus S., Vėjelis S., Girskas G., Balčiūnas G. (2018). Rapeseed-Based Polyols and Paper Production Waste Sludge in Polyurethane Foam: Physical Properties and Their Prediction Models. Ind. Crops Prod..

[43] Mills N.J. (2005). The Wet Kelvin Model for Air Flow through Open-Cell Polyurethane Foams. J. Mater. Sci..

[44] Ionescu M. (2005). Chemistry and Technology of Polyols for Polyurethanes.

[45] Hejna A. (2021). Clays as Inhibitors of Polyurethane Foams’ Flammability. Materials.

[46] Ates M., Karadag S., Eker A.A., Eker B. (2022). Polyurethane Foam Materials and Their Industrial Applications. Polym. Int..

[47] Fagnani D.E., Tami J.L., Copley G., Clemons M.N., Getzler Y.D.Y.L., McNeil A.J. (2021). 100th Anniversary of Macromolecular Science Viewpoint: Redefining Sustainable Polymers. ACS Macro Lett..

[48] Hong M., Chen E.Y.X. (2019). Future Directions for Sustainable Polymers. Trends Chem..

[49] Polyurethane Market|Global Research Report 2032. https://www.factmr.com/report/polyurethane-market.

[50] Peyrton J., Avérous L. (2021). Structure-Properties Relationships of Cellular Materials from Biobased Polyurethane Foams. Mater. Sci. Eng. R Rep..

[51] Engels H.W., Pirkl H.G., Albers R., Albach R.W., Krause J., Hoffmann A., Casselmann H., Dormish J. (2013). Polyurethanes: Versatile Materials and Sustainable Problem Solvers for Today’s Challenges. Angew. Chem. Int. Ed..

[52] Furtwengler P., Avérous L. (2018). Renewable Polyols for Advanced Polyurethane Foams from Diverse Biomass Resources. Polym. Chem..

[53] Laurichesse S., Avérous L. (2014). Chemical Modification of Lignins: Towards Biobased Polymers. Prog. Polym. Sci..

[54] Desroches M., Escouvois M., Auvergne R., Caillol S., Boutevin B. (2012). From Vegetable Oils to Polyurethanes: Synthetic Routes to Polyols and Main Industrial Products. Polym. Rev..

[55] Fridrihsone A., Romagnoli F., Kirsanovs V., Cabulis U. (2020). Life Cycle Assessment of Vegetable Oil Based Polyols for Polyurethane Production. J. Clean. Prod..

[56] Sahoo S.K., Khandelwal V., Manik G. (2018). Development of Completely Bio-based Epoxy Networks Derived from Epoxidized Linseed and Castor Oil Cured with Citric Acid. Polym. Adv. Technol..

[57] Khandelwal V., Sahoo S.K., Kumar A., Sethi S.K., Manik G. (2019). Bio-Sourced Electrically Conductive Epoxidized Linseed Oil Based Composites Filled with Polyaniline and Carbon Nanotubes. Compos. B Eng..

[58] Singh I., Samal S.K., Mohanty S., Nayak S.K. (2020). Recent Advancement in Plant Oil Derived Polyol-Based Polyurethane Foam for Future Perspective: A Review. Eur. J. Lipid Sci. Technol..

[59] Tu Y., Kiatsimkul P., Suppes G., Hsieh F. (2007). Physical Properties of Water-blown Rigid Polyurethane Foams from Vegetable Oil-based Polyols. J. Appl. Polym. Sci..

[60] Guo A., Zhang W., Petrovic Z.S. (2006). Structure–Property Relationships in Polyurethanes Derived from Soybean Oil. J. Mater. Sci..

[61] Petrović Z.S., Zhang W., Javni I. (2005). Structure and Properties of Polyurethanes Prepared from Triglyceride Polyols by Ozonolysis. Biomacromolecules.

[62] Tan S., Abraham T., Ference D., MacOsko C.W. (2011). Rigid Polyurethane Foams from a Soybean Oil-Based Polyol. Polymer.

[63] Tu Y.C., Fan H., Suppes G.J., Hsieh F.H. (2009). Physical Properties of Water-Blown Rigid Polyurethane Foams Containing Epoxidized Soybean Oil in Different Isocyanate Indices. J. Appl. Polym. Sci..

[64] Ji D., Fang Z., He W., Luo Z., Jiang X., Wang T., Guo K. (2015). Polyurethane Rigid Foams Formed from Different Soy-Based Polyols by the Ring Opening of Epoxidised Soybean Oil with Methanol, Phenol, and Cyclohexanol. Ind. Crops Prod..

[65] Narine S.S., Kong X., Bouzidi L., Sporns P. (2006). Physical Properties of Polyurethanes Produced from Polyols from Seed Oils: II. Foams. J. Am. Oil Chem. Soc..

[66] Kurańska M., Prociak A. (2016). The Influence of Rapeseed Oil-Based Polyols on the Foaming Process of Rigid Polyurethane Foams. Ind. Crops Prod..

[67] Zieleniewska M., Leszczyński M.K., Kurańska M., Prociak A., Szczepkowski L., Krzyzowska M., Ryszkowska J. (2015). Preparation and Characterisation of Rigid Polyurethane Foams Using a Rapeseed Oil-Based Polyol. Ind. Crops Prod..

[68] Uram K., Prociak A., Kurańska M. (2020). Influence of the Chemical Structure of Rapeseed Oil-Based Polyols on Selected Properties of Polyurethane Foams. Polimery.

[69] Kurańska M., Pinto J.A., Salach K., Barreiro M.F., Prociak A. (2020). Synthesis of Thermal Insulating Polyurethane Foams from Lignin and Rapeseed Based Polyols: A Comparative Study. Ind. Crops Prod..

[70] Kairytė A., Vaitkus S., Vėjelis S., Pundienė I. (2018). A Study of Rapeseed Oil-Based Polyol Substitution with Bio-Based Products to Obtain Dimensionally and Structurally Stable Rigid Polyurethane Foam. J. Polym. Environ..

[71] Ogunniyi D.S. (2006). Castor Oil: A Vital Industrial Raw Material. Bioresour. Technol..

[72] Zhang M., Pan H., Zhang L., Hu L., Zhou Y. (2014). Study of the Mechanical, Thermal Properties and Flame Retardancy of Rigid Polyurethane Foams Prepared from Modified Castor-Oil-Based Polyols. Ind. Crops Prod..

[73] Ionescu M., Radojčić D., Wan X., Shrestha M.L., Petrović Z.S., Upshaw T.A. (2016). Highly Functional Polyols from Castor Oil for Rigid Polyurethanes. Eur. Polym. J..

[74] Carriço C.S., Fraga T., Carvalho V.E., Pasa V.M.D. (2017). Polyurethane Foams for Thermal Insulation Uses Produced from Castor Oil and Crude Glycerol Biopolyols. Molecules.

[75] Hejna A., Kirpluks M., Kosmela P., Cabulis U., Haponiuk J., Piszczyk Ł. (2017). The Influence of Crude Glycerol and Castor Oil-Based Polyol on the Structure and Performance of Rigid Polyurethane-Polyisocyanurate Foams. Ind. Crops Prod..

[76] Arniza M.Z., Hoong S.S., Idris Z., Yeong S.K., Hassan H.A., Din A.K., Choo Y.M. (2015). Synthesis of Transesterified Palm Olein-Based Polyol and Rigid Polyurethanes from This Polyol. JAOCS J. Am. Oil Chem. Soc..

[77] Srihanum A., Tuan Noor M.T.I., Devi K.P.P., Hoong S.S., Ain N.H., Mohd N.S., Nek Mat Din N.S.M., Kian Y.S. (2022). Low Density Rigid Polyurethane Foam Incorporated with Renewable Polyol as Sustainable Thermal Insulation Material. J. Cell. Plast..

[78] Marcovich N.E., Kurańska M., Prociak A., Malewska E., Kulpa K. (2017). Open Cell Semi-Rigid Polyurethane Foams Synthesized Using Palm Oil-Based Bio-Polyol. Ind. Crops Prod..

[79] Jaratrotkamjorn R., Tanrattanakul V. (2020). Bio-based Flexible Polyurethane Foam Synthesized from Palm Oil and Natural Rubber. J. Appl. Polym. Sci..

[80] Paciorek-Sadowska J., Borowicz M., Czupryński B., Isbrandt M. (2018). Effect of Evening Primrose Oil-Based Polyol on the Properties of Rigid Polyurethane–Polyisocyanurate Foams for Thermal Insulation. Polymers.

[81] Ionescu M., Petrović Z.S. (2010). High Functionality Polyether Polyols Based on Polyglycerol. J. Cell. Plast..

[82] Luo X., Hu S., Zhang X., Li Y. (2013). Thermochemical Conversion of Crude Glycerol to Biopolyols for the Production of Polyurethane Foams. Bioresour. Technol..

[83] Paruzel A., Michałowski S., Hodan J., Horák P., Prociak A., Beneš H. (2017). Rigid Polyurethane Foam Fabrication Using Medium Chain Glycerides of Coconut Oil and Plastics from End-of-Life Vehicles. ACS Sustain. Chem. Eng..

[84] Arbenz A., Perrin R., Avérous L. (2018). Elaboration and Properties of Innovative Biobased PUIR Foams from Microalgae. J. Polym. Environ..

[85] Pawar M.S., Kadam A.S., Dawane B.S., Yemul O.S. (2016). Synthesis and Characterization of Rigid Polyurethane Foams from Algae Oil Using Biobased Chain Extenders. Polym. Bull..

[86] Peyrton J., Chambaretaud C., Sarbu A., Avérous L. (2020). Biobased Polyurethane Foams Based on New Polyol Architectures from Microalgae Oil. ACS Sustain. Chem. Eng..

[87] Kosmela P., Kazimierski P., Formela K., Haponiuk J., Piszczyk Ł. (2017). Liquefaction of Macroalgae Enteromorpha Biomass for the Preparation of Biopolyols by Using Crude Glycerol. J. Ind. Eng. Chem..

[88] Petrović Z.S., Wan X., Bilić O., Zlatanić A., Hong J., Javni I., Ionescu M., Milić J., Degruson D. (2013). Polyols and Polyurethanes from Crude Algal Oil. JAOCS J. Am. Oil Chem. Soc..

[89] Prociak A., Kurańska M., Cabulis U., Ryszkowska J., Leszczyńska M., Uram K., Kirpluks M. (2018). Effect of Bio-Polyols with Different Chemical Structures on Foaming of Polyurethane Systems and Foam Properties. Ind. Crops Prod..

[90] Paciorek-Sadowska J., Borowicz M., Czupryński B., Tomaszewska E., Liszkowska J. (2018). Oenothera Biennis Seed Oil as an Alternative Raw Material for Production of Bio-Polyol for Rigid Polyurethane-Polyisocyanurate Foams. Ind. Crops Prod..

[91] Huang X., De Hoop C.F., Xie J., Wu Q., Boldor D., Qi J. (2018). High Bio-Content Polyurethane (PU) Foam Made from Bio-Polyol and Cellulose Nanocrystals (CNCs) via Microwave Liquefaction. Mater. Des..

[92] Ma Y., Xiao Y., Zhao Y., Bei Y., Hu L., Zhou Y., Jia P. (2022). Biomass Based Polyols and Biomass Based Polyurethane Materials as a Route towards Sustainability. React. Funct. Polym..

[93] Kim J.Y., Lee H.W., Lee S.M., Jae J., Park Y.K. (2019). Overview of the Recent Advances in Lignocellulose Liquefaction for Producing Biofuels, Bio-Based Materials and Chemicals. Bioresour. Technol..

[94] Szpiłyk M., Lubczak R., Lubczak J. (2021). The Biodegradable Cellulose-Derived Polyol and Polyurethane Foam. Polym. Test..

[95] Nadji H., Bruzzèse C., Belgacem M.N., Benaboura A., Gandini A. (2005). Oxypropylation of Lignins and Preparation of Rigid Polyurethane Foams from the Ensuing Polyols. Macromol. Mater. Eng..

[96] Jiang D., Wang Y., Li B., Sun C., Guo Z. (2020). Environmentally Friendly Alternative to Polyester Polyol by Corn Straw on Preparation of Rigid Polyurethane Composite. Compos. Commun..

[97] D’Souza J., Camargo R., Yan N. (2014). Polyurethane Foams Made from Liquefied Bark-Based Polyols. J. Appl. Polym. Sci..

[98] Xie J., Zhai X., Hse C.Y., Shupe T.F., Pan H. (2015). Polyols from Microwave Liquefied Bagasse and Its Application to Rigid Polyurethane Foam. Materials.

[99] Yue D., Oribayo O., Rempel G.L., Pan Q. (2017). Liquefaction of Waste Pine Wood and Its Application in the Synthesis of a Flame Retardant Polyurethane Foam. RSC Adv..

[100] Fidan M.S., Ertaş M. (2020). Biobased Rigid Polyurethane Foam Prepared from Apricot Stone Shell-Based Polyol for Thermal Insulation Application, Part 1: Synthesis, Chemical, and Physical Properties. Bioresources.

[101] Huang G., Wang P. (2017). Effects of Preparation Conditions on Properties of Rigid Polyurethane Foam Composites Based on Liquefied Bagasse and Jute Fibre. Polym. Test..

[102] Esteves B., Dulyanska Y., Costa C., Vicente J., Domingos I., Pereira H., de Lemos L.T., Cruz-Lopes L. (2017). Cork Liquefaction for Polyurethane Foam Production. Bioresources.

[103] Wang Q., Tuohedi N. (2020). Polyurethane Foams and Bio-Polyols from Liquefied Cotton Stalk Agricultural Waste. Sustainability.

[104] Xu J., Jiang J., Hse C.Y., Shupe T.F. (2014). Preparation of Polyurethane Foams Using Fractionated Products in Liquefied Wood. J. Appl. Polym. Sci..

[105] Li H.Q., Shao Q., Luo H., Xu J. (2016). Polyurethane Foams from Alkaline Lignin-Based Polyether Polyol. J. Appl. Polym. Sci..

[106] Arbenz A., Frache A., Cuttica F., Avérous L. (2016). Advanced Biobased and Rigid Foams, Based on Urethane-Modified Isocyanurate from Oxypropylated Gambier Tannin Polyol. Polym. Degrad. Stab..

[107] Kosmela P., Hejna A., Formela K., Haponiuk J.T., Piszczyk Ł. (2016). Biopolyols Obtained via Crude Glycerol-Based Liquefaction of Cellulose: Their Structural, Rheological and Thermal Characterization. Cellulose.

[108] Soares B., Gama N., Freire C.S.R., Barros-Timmons A., Brandão I., Silva R., Neto C.P., Ferreira A. (2015). Spent Coffee Grounds as a Renewable Source for Ecopolyols Production. J. Chem. Technol. Biotechnol..

[109] Domingos I.J., Fernandes A.P., Ferreira J., Cruz-Lopes L., Esteves B.M. (2019). Polyurethane Foams from Liquefied Eucalyptus Globulus Branches. Bioresources.

[110] Kosmela P., Hejna A., Suchorzewski J., Piszczyk Ł., Haponiuk J.T. (2020). Study on the Structure-Property Dependences of Rigid PUR-PIR Foams Obtained from Marine Biomass-Based Biopolyol. Materials.

[111] Ionescu M., Wan X., Bilić N., Petrović Z.S. (2012). Polyols and Rigid Polyurethane Foams from Cashew Nut Shell Liquid. J. Polym. Environ..

[112] Gandhi T.S., Patel M.R., Dholakiya B.Z. (2015). Mechanical, Thermal and Fire Properties of Sustainable Rigid Polyurethane Foam Derived from Cashew Nut Shell Liquid. Int. J. Plast. Technol..

[113] De Luca Bossa F., Santillo C., Verdolotti L., Campaner P., Minigher A., Boggioni L., Losio S., Coccia F., Iannace S., Lama G.C. (2020). Greener Nanocomposite Polyurethane Foam Based on Sustainable Polyol and Natural Fillers: Investigation of Chemico-Physical and Mechanical Properties. Materials.

[114] Zhang J., Hori N., Takemura A. (2019). Optimization of Agricultural Wastes Liquefaction Process and Preparing Bio-Based Polyurethane Foams by the Obtained Polyols. Ind. Crops Prod..

[115] Zhang X., Kim Y., Elsayed I., Taylor M., Eberhardt T.L., Hassan E.B., Shmulsky R. (2019). Rigid Polyurethane Foams Containing Lignin Oxyalkylated with Ethylene Carbonate and Polyethylene Glycol. Ind. Crops Prod..

[116] Duval A., Vidal D., Sarbu A., René W., Avérous L. (2022). Scalable Single-Step Synthesis of Lignin-Based Liquid Polyols with Ethylene Carbonate for Polyurethane Foams. Mater. Today Chem..

[117] Hatakeyama H., Kosugi R., Hatakeyama T. (2008). Thermal Properties of Lignin-and Molasses-Based Polyurethane Foams. J. Therm. Anal. Calorim..

[118] Gao L.L., Liu Y.H., Lei H., Peng H., Ruan R. (2010). Preparation of Semirigid Polyurethane Foam with Liquefied Bamboo Residues. J. Appl. Polym. Sci..

[119] Hu S., Li Y. (2014). Two-Step Sequential Liquefaction of Lignocellulosic Biomass by Crude Glycerol for the Production of Polyols and Polyurethane Foams. Bioresour. Technol..

[120] Li B., Zhou M., Huo W., Cai D., Qin P., Cao H., Tan T. (2020). Fractionation and Oxypropylation of Corn-Stover Lignin for the Production of Biobased Rigid Polyurethane Foam. Ind. Crops Prod..

[121] Hwang U., Lee B., Oh B., Shin H.S., Lee S.S., Kang S.G., Kim D., Park J., Shin S., Suhr J. (2022). Hydrophobic Lignin/Polyurethane Composite Foam: An Eco-Friendly and Easily Reusable Oil Sorbent. Eur. Polym. J..

[122] Kuranchie C., Yaya A., Bensah Y.D. (2021). The Effect of Natural Fibre Reinforcement on Polyurethane Composite Foams—A Review. Sci. Afr..

[123] Pickering K.L., Efendy M.G.A., Le T.M. (2016). A Review of Recent Developments in Natural Fibre Composites and Their Mechanical Performance. Compos. Part A Appl. Sci. Manuf..

[124] Zhou X., Sain M.M., Oksman K. (2016). Semi-Rigid Biopolyurethane Foams Based on Palm-Oil Polyol and Reinforced with Cellulose Nanocrystals. Compos. Part A Appl. Sci. Manuf..

[125] Zhou X., Sethi J., Geng S., Berglund L., Frisk N., Aitomäki Y., Sain M.M., Oksman K. (2016). Dispersion and Reinforcing Effect of Carrot Nanofibers on Biopolyurethane Foams. Mater. Des..

[126] Xue B.L., Wen J.L., Sun R.C. (2014). Lignin-Based Rigid Polyurethane Foam Reinforced with Pulp Fiber: Synthesis and Characterization. ACS Sustain. Chem. Eng..

[127] Ribeiro Da Silva V., Mosiewicki M.A., Yoshida M.I., Coelho Da Silva M., Stefani P.M., Marcovich N.E. (2013). Polyurethane Foams Based on Modified Tung Oil and Reinforced with Rice Husk Ash II: Mechanical Characterization. Polym. Test..

[128] Paberza A., Cabulis U., Arshanitsa A. (2014). Wheat Straw Lignin as Filler for Rigid Polyurethane Foams on the Basis of Tall Oil Amide. Polimery.

[129] Zieleniewska M., Leszczyński M.K., Szczepkowski L., Bryśkiewicz A., Krzyżowska M., Bień K., Ryszkowska J. (2016). Development and Applicational Evaluation of the Rigid Polyurethane Foam Composites with Egg Shell Waste. Polym. Degrad. Stab..

[130] Silva M.C., Takahashi J.A., Chaussy D., Belgacem M.N., Silva G.G. (2010). Composites of Rigid Polyurethane Foam and Cellulose Fiber Residue. J. Appl. Polym. Sci..

[131] Prociak A., Kurañska M., Malewska E., Szczepkowski L., Zieleniewska M., Ryszkowska J., Ficon J., Rzasa A. (2015). Biobased Polyurethane Foams Modified with Natural Fillers. Polimery.

[132] Mosiewicki M.A., Dell’Arciprete G.A., Aranguren M.I., Marcovich N.E. (2009). Polyurethane Foams Obtained from Castor Oil-Based Polyol and Filled with Wood Flour. J. Compos. Mater..

[133] Joanna P.S., Bogusław C., Joanna L. (2011). Application of Waste Products from Agricultural-Food Industry for Production of Rigid Polyurethane-Polyisocyanurate Foams. J. Porous Mater..

[134] Qiu Q., Yang X., Zhang P., Wang D., Lu M., Wang Z., Guo G., Yu J., Tian H., Li J. (2021). Effect of Fiber Surface Treatment on the Structure and Properties of Rigid Bagasse Fibers/Polyurethane Composite Foams. Polym. Compos..

[135] de Avila Delucis R., Magalhães W.L.E., Petzhold C.L., Amico S.C. (2018). Forest-Based Resources as Fillers in Biobased Polyurethane Foams. J. Appl. Polym. Sci..

[136] Jabber L.J.Y., Grumo J.C., Alguno A.C., Lubguban A.A., Capangpangan R.Y. (2021). Influence of Cellulose Fibers Extracted from Pineapple (Ananas Comosus) Leaf to the Mechanical Properties of Rigid Polyurethane Foam. Mater. Today Proc..

[137] Uram K., Kurańska M., Andrzejewski J., Prociak A. (2021). Rigid Polyurethane Foams Modified with Biochar. Materials.

[138] Uram K., Leszczyńska M., Prociak A., Czajka A., Gloc M., Leszczyński M.K., Michałowski S., Ryszkowska J. (2021). Polyurethane Composite Foams Synthesized Using Bio-Polyols and Cellulose Filler. Materials.

[139] Kuźnia M., Magiera A., Zygmunt-Kowalska B., Kaczorek-Chrobak K., Pielichowska K., Szatkowski P., Benko A., Ziabka M., Jerzak W. (2021). Fly Ash as an Eco-Friendly Filler for Rigid Polyurethane Foams Modification. Materials.

[140] Paciorek-Sadowska J., Czupryński B., Borowicz M., Liszkowska J. (2020). Rigid Polyurethane–Polyisocyanurate Foams Modified with Grain Fraction of Fly Ashes. J. Cell. Plast..

[141] Liszkowska J. (2018). The Effect of Ground Coffee on the Mechanical and Application Properties of Rigid Polyurethane-Polyisocyanurate Foams. Polimery.

[142] Wrześniewska-Tosik K., Ryszkowska J., Mik T., Wesołowska E., Kowalewski T., Pałczyńska M., Sałasińska K., Walisiak D., Czajka A. (2020). Composites of Semi-Rigid Polyurethane Foams with Keratin Fibers Derived from Poultry Feathers and Flame Retardant Additives. Polymers.

[143] Kuranska M., Prociak A., Michalowski S., Cabulis U., Kirpluks M. (2016). Microcellulose as a Natural Filler in Polyurethane Foams Based on the Biopolyol from Rapeseed Oil. Polimery.

[144] Luo X., Mohanty A., Misra M. (2012). Water-Blown Rigid Biofoams from Soy-Based Biopolyurethane and Microcrystalline Cellulose. JAOCS J. Am. Oil Chem. Soc..

[145] Tian H., Wu J., Xiang A. (2018). Polyether Polyol-Based Rigid Polyurethane Foams Reinforced with Soy Protein Fillers. J. Vinyl Addit. Technol..

[146] Khaleel M., Soykan U., Çetin S. (2021). Influences of Turkey Feather Fiber Loading on Significant Characteristics of Rigid Polyurethane Foam: Thermal Degradation, Heat Insulation, Acoustic Performance, Air Permeability and Cellular Structure. Constr. Build. Mater..

[147] Sair S., Oushabi A., Kammouni A., Tanane O., Abboud Y., El Bouari A. (2018). Mechanical and Thermal Conductivity Properties of Hemp Fiber Reinforced Polyurethane Composites. Case Stud. Constr. Mater..

[148] de Avila Delucis R., Fischer Kerche E., Gatto D.A., Magalhães Esteves W.L., Petzhold C.L., Campos Amico S. (2019). Surface Response and Photodegradation Performance of Bio-Based Polyurethane-Forest Derivatives Foam Composites. Polym. Test..

[149] Oushabi A., Sair S., Abboud Y., Tanane O., Bouari A. (2017). El An Experimental Investigation on Morphological, Mechanical and Thermal Properties of Date Palm Particles Reinforced Polyurethane Composites as New Ecological Insulating Materials in Building. Case Stud. Constr. Mater..

[150] Husainie S.M., Deng X., Ghalia M.A., Robinson J., Naguib H.E. (2021). Natural Fillers as Reinforcement for Closed-Molded Polyurethane Foam Plaques: Mechanical, Morphological, and Thermal Properties. Mater. Today Commun..

[151] Kairytė A., Kizinievič O., Kizinievič V., Kremensas A. (2019). Synthesis of Biomass-Derived Bottom Waste Ash Based Rigid Biopolyurethane Composite Foams: Rheological Behaviour, Structure and Performance Characteristics. Compos. Part A Appl. Sci. Manuf..

[152] Husainie S.M., Khattak S.U., Robinson J., Naguib H.E. (2020). A Comparative Study on the Mechanical Properties of Different Natural Fiber Reinforced Free-Rise Polyurethane Foam Composites. Ind. Eng. Chem. Res..

[153] Kairytė A., Członka S., Boris R., Vėjelis S. (2021). Vacuum-Based Impregnation of Liquid Glass into Sunflower Press Cake Particles and Their Use in Bio-Based Rigid Polyurethane Foam. Materials.

[154] Głowacz-Czerwonka D., Zakrzewska P., Oleksy M., Pielichowska K., Kuźnia M., Telejko T. (2023). The Influence of Biowaste-Based Fillers on the Mechanical and Fire Properties of Rigid Polyurethane Foams. Sustain. Mater. Technol..

[155] Anwar M.F., Yu L.J., Lim Y.M., Tarawneh M.A., Se Yong E.N., Lai N.Y.G. (2023). Water Absorption Properties of Polyurethane Foam Reinforced with Paper Pulp. Mater. Today Proc..

[156] Chanlert P., Ruamcharoen P. (2021). Sound Absorption Properties of Rigid Polyurethane Foam Composites with Rubber-Wood Sawdust as a Natural Filler. J. Phys. Conf. Ser..

[157] Olcay H., Kocak E.D. (2022). The Mechanical, Thermal and Sound Absorption Properties of Flexible Polyurethane Foam Composites Reinforced with Artichoke Stem Waste Fibers. J. Ind. Text..

[158] Olcay H., Kocak E.D. (2021). Rice Plant Waste Reinforced Polyurethane Composites for Use as the Acoustic Absorption Material. Appl. Acoust..

[159] Ekici B., Kentli A., Küçük H. (2012). Improving Sound Absorption Property of Polyurethane Foams by Adding Tea-Leaf Fibers. Arch. Acoust..

[160] Polyurethane Market Size & Share Analysis—Growth Trends & Forecasts (2023–2028). https://www.mordorintelligence.com/industry-reports/polyurethane-market.

[161] Yang W., Dong Q., Liu S., Xie H., Liu L., Li J. (2012). Recycling and Disposal Methods for Polyurethane Foam Wastes. Procedia Environ. Sci..

[162] Deng Y., Dewil R., Appels L., Ansart R., Baeyens J., Kang Q. (2021). Reviewing the Thermo-Chemical Recycling of Waste Polyurethane Foam. J. Environ. Manag..

[163] Cregut M., Bedas M., Durand M.-J., Thouand G. (2013). New Insights into Polyurethane Biodegradation and Realistic Prospects for the Development of a Sustainable Waste Recycling Process. Biotechnol. Adv..

[164] Howard G.T. (2011). Microbial Biodegradation of Polyurethane in: Polyurethane Biodegradation.

[165] Rossignolo G., Malucelli G., Lorenzetti A. (2024). Recycling of Polyurethanes: Where We Are and Where We Are Going. Green Chem..

[166] Liu B., Westman Z., Richardson K., Lim D., Stottlemyer A.L., Farmer T., Gillis P., Hooshyar N., Vlcek V., Christopher P. (2024). Polyurethane Foam Chemical Recycling: Fast Acidolysis with Maleic Acid and Full Recovery of Polyol. ACS Sustain. Chem. Eng..

[167] Zia K.M., Bhatti H.N., Ahmad Bhatti I. (2007). Methods for Polyurethane and Polyurethane Composites, Recycling and Recovery: A Review. React. Funct. Polym..

[168] Sternberg J., Pilla S. (2023). Chemical Recycling of a Lignin-Based Non-Isocyanate Polyurethane Foam. Nat. Sustain..

[169] Gama N.V., Ferreira A., Barros-Timmons A. (2018). Polyurethane Foams: Past, Present, and Future. Materials.

[170] Gómez-Rojo R., Alameda L., Rodríguez Á., Calderón V., Gutiérrez-González S. (2019). Characterization of Polyurethane Foam Waste for Reuse in Eco-Efficient Building Materials. Polymers.

[171] Kemona A., Piotrowska M. (2020). Polyurethane Recycling and Disposal: Methods and Prospects. Polymers.

[172] Magnin A., Entzmann L., Bazin A., Pollet E., Avérous L. (2021). Green Recycling Process for Polyurethane Foams by a Chem-Biotech Approach. ChemSusChem.

[173] Liu J., Liu J., Xu B., Xu A., Cao S., Wei R., Zhou J., Jiang M., Dong W. (2022). Biodegradation of Polyether-Polyurethane Foam in Yellow Mealworms (Tenebrio Molitor) and Effects on the Gut Microbiome. Chemosphere.

[174] Gunawan N.R., Tessman M., Zhen D., Johnson L., Evans P., Clements S.M., Pomeroy R.S., Burkart M.D., Simkovsky R., Mayfield S.P. (2022). Biodegradation of Renewable Polyurethane Foams in Marine Environments Occurs through Depolymerization by Marine Microorganisms. Sci. Total Environ..

